# PBRM1 presents a potential prognostic marker and therapeutic target in duodenal papillary carcinoma

**DOI:** 10.1002/ctm2.1062

**Published:** 2022-09-30

**Authors:** Xujun He, Ji Xu, Nan Niu, Guoxi Xu, Honglin Zhu, Zhengchuang Liu, Yiping Mou, Zhengyuan Qian, Huiju Wang, Junfeng Hu, Tonghui Ma, Jie Ma, Houquan Tao

**Affiliations:** ^1^ Key Laboratory of Gastroenterology of Zhejiang Province Zhejiang Provincial People's Hospital (Affiliated People's Hospital Hangzhou Medical College) Hangzhou Zhejiang China; ^2^ Department of Genetic and Genome Medicine Zhejiang Provincial People's Hospital (Affiliated People's Hospital, Hangzhou Medical College) Hangzhou Zhejiang China; ^3^ Department of Gastrointestinal and Pancreatic Surgery Zhejiang Provincial People's Hospital (Affiliated People's Hospital, Hangzhou Medical College) Hangzhou Zhejiang China; ^4^ The Second Clinical Medical College of Zhejiang Chinese Medical University Hangzhou Zhejiang China; ^5^ Department of Gastrointestinal Surgery Jinjiang Hospital Quanzhou Fujian China; ^6^ Genetron Health (Beijing) Technology Co. Ltd. Beijing China; ^7^ Department of Pathology Zhejiang Provincial People's Hospital (Affiliated People's Hospital, Hangzhou Medical College) Hangzhou Zhejiang China

**Keywords:** duodenal papillary carcinoma, genomic profiling, mutation, PBRM1

## Abstract

**Background:**

Due to its rarity, duodenal papillary carcinoma (DPC) is seldom studied as a unique disease and no specific molecular features or treatment guidelines are provided.

**Methods:**

Whole‐exome sequencing was performed to gain new insights into the DPC mutation landscape and to identify potential signalling pathways and therapeutic targets. Mechanistically, immunohistochemistry (IHC), immunofluorescence, RNA‐seq, ATAC‐seq and in vitro cell function experiments were performed to confirm the underlying mechanisms.

**Results:**

We described the mutational landscape of DPC for the first time as a group of rare tumours with a high frequency of dysregulation in the chromatin remodelling pathway, particularly PBRM1‐inactivating mutations that are significantly higher than duodenal adenocarcinomas and ampullary adenocarcinoma (27% vs. 0% vs. 7%, *p <* *.01*). In vitro cell experiments showed that downregulation of PBRM1 expression could significantly promote the cancer progression and epithelial‐to‐mesenchymal transition via the PBRM1‐c‐JUN‐VIM axis. The IHC data indicated that PBRM1 deficiency (*p = .047*) and c‐JUN expression (*p <* *.001*) were significantly associated with poor prognosis. Meanwhile, the downregulation of PBRM1 expression in HUTU‐80 cells was sensitive to radiation, which may be due to the suppression of c‐JUN by irradiation.

**Conclusions:**

Our findings define a novel molecular subgroup of PBRM1‐inactivating mutations in DPC. PBRM1 play an important role in DPC progression and may serve as a potential therapeutic target and prognostic indicator.

## Graphical abstract

We identified frequent mutation in the gene of PBRM1 which involved in the chromatin remodeling pathway and may represent a molecular subgroup in DPC. PBRM1 play an important role in DPC progression and may serve as a potential prognostic marker and therapeutic target.

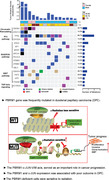

## 1 INTRODUCTION

The ampulla, also known as the biliopancreatic duodenal junction area, includes the lower end of the common bile duct, the distal end of the pancreatic duct, the duodenal papilla and the surrounding 2‐cm area. Thus, this complex cellular environment from the ampulla can form a group of histopathological heterogeneous tumours, such as carcinomas of the ampulla of Vater (VAC), lower segment carcinoma of the bile duct (BDC), duodenal papillary carcinoma (DPC), and pancreatic head carcinoma (PHC).^1^ Accurate diagnosis of these carcinomas is challenging due to their histologic similarities and the adjacent anatomical location. Meanwhile, because accurate preoperative diagnosis is sometimes elusive for these tumours, surgical resection by pancreaticoduodenectomy and conventional chemotherapy offer the only chance for a cure. DPC is a type of primary tumour of the main duodenal papilla area and belongs to the category of periampullary carcinoma. It is a rare neoplasm, accounting for only 0.01% of malignant tumours and less than 1% of all malignancies of the digestive system.^2^ However, DPC is the second most common periampullary malignancy, second to PHC. It has been reported that among malignant tumours that primarily occur in the duodenum, 60% are diagnosed as DPC, and the trend is increasing year by year.^3^ Most DPCs are diagnosed at late stages, and surgery is the only effective treatment option. Even for those patients receiving surgery with curative intent, the 5‐year survival rate is only approximately 46%.^4^ Due to their rarity and the challenges of diagnosis, understanding of the molecular features of these tumours is limited.

Previous large‐scale studies on small bowel adenocarcinoma, ampullary adenocarcinoma (AMPAC) and duodenal adenocarcinoma (DUOAC) were mostly focused on known cancer genes.^5^, ^6^, ^7^, ^8^, ^9^ Similar to colorectal cancer (CRC), the top mutated genes in small bowel adenocarcinoma, AMPAC and DUOAC are *TP53, KRAS, APC* and *SMAD4*, but with different frequency rates. Additionally, they found that *ELF3* is a driver in AMPAC and that AMPAC exhibited frequent Wnt dysregulation. Meanwhile, DUOAC and other small bowel cancers also harbour *ERBB2* amplifications, a targetable molecular feature in gastric and CRCs. There are also differences in mutational profiles among small bowel cancers with different locations.^5^ Nevertheless, to our knowledge, no studies have focused specifically on the molecular features of DPC, and a limited number of known unique prognostic markers and molecular features have been identified for DPC. Therefore, given the clinical need for better markers for the diagnosis and treatment of DPC, we sought to elucidate the pattern of somatic mutations to achieve a more comprehensive view of the molecular landscape of DPC. In this study, we performed whole‐exome sequencing of 15 DPCs to gain new insights into DPC biology. In addition, IHC, Immunofluorescence (IF), RNA‐seq, ATAC‐seq and in vitro cell function experiments were used to investigate the mechanisms, in order to identify potential signalling pathways and therapeutic targets.

## 2 METHOD AND MATERIALS

### 2.1 Discovery cohort (next‐generation sequencing sample)

15 DPC tissues and matched blood samples were collected for whole‐exon sequencing (WES). All patients underwent surgical resection and were followed up (Sheet S1).

### 2.2 Validation cohort (IHC and follow‐up)

A total of 116 DPC patients were enrolled from January 2008 to March 2015 in Zhejiang Provincial People's Hospital, with a median age of 62.8 years (range: 17‐89 years). The median follow‐up duration was 44.6 months (1‐132 months), with the 5‐year and 3‐year survival rates being 45% and 51%, respectively. All DPC cases were classified according to the 8th Edition of Ampulla Cancer by American Joint Commission on Cancer (AJCC). In Table S1, we summarize the clinicopathological characteristics of the patients.

### 2.3 Whole exon sequencing and data analysis

Genomic DNA was extracted using the QIAamp DNA Tissue & Blood Kit (Qiagen) and fragmented to 150‐200 bp with an M220 Focused‐ultrasonicator (Covaris). Libraries were prepared with KAPA HTP Library Preparation Kit and captured with the Agilent SureSelect V5 system. Sequencing was performed on HiSeq X Ten (Illumina) at Genetron Health. Co., Ltd. Then we used Trimmomatic (v0.36) to remove adapters and low‐quality reads and mapped the filtered reads to the human genome (hg19) with BWA (v0.7.10). Single‐nucleotide variants (SNV) and insertion/deletions (InDels) were detected using muTect^10^ and Strelka,^11^ respectively. Copy number variations (CNVs) and structural variation (SV) were identified using ADTEx^12^ and CREST,^13^ respectively. All mutations were annotated using Oncotator^14^ and Vep.^15^ KOBAS 3.0^16^ was used for KEGG enrichment analysis.

### 2.4 Mutational signatures

Somatic mutations across the whole exome data were used to analyse mutational spectrums and signatures. First, we calculated the proportions of six types of substitution: C > T/G > A, C > G/G > C, C > A/G > T, T > G/A > C, T > C/A > G, T > A/A > T, and carried out clustering analysis by using the proportional results. Furthermore, signatures of 96‐substitution classifications defined by the substitution context of flanking bases were extracted following the non‐negative matrix factorization (NMF) algorithm.^17^ At the last, the extracted signatures were compared with previously reported signatures archived in the COSMIC database (https://cosmic‐blog.sanger.ac.uk), in terms of cosine similarity.

### 2.5 Cell culture and treatment

Human DUOAC HUTU‐80 cell and breast cancer cell MDA‐MB‐231 were purchased from the Cell Resource Center of the Chinese Academy of Medical Sciences. HUTU‐80 cells were cultured in Minimum Essential Medium (MEM) with 10% foetal bovine serum (FBS) and 1% non‐essential amino acid (NEAA). MDA‐MB‐231 cells were maintained in a 10% FBS DMEM medium.

### 2.6 Generation of cell lines

Knockdown of PBRM1 expression was performed using lentiviral construct pLKO.1 and packaged as Lentiviral by Guanzhou RIBOBIO Co., Ltd. The PBRM1 shRNA mature antisense sequences are: shRNA #1 GGAAGATGCTACAGCGATT, shRNA #2 GTACCAAGATATTGACTCT and shRNA #3 GAACCAGGTTGCCACTACT. The shNTC cell was transfected with a non‐targeting hairpin and the non‐targeting sequence is CAACAAGATGAAGAGCACCAA. The NTC cell was transfected with an empty vector. The HUTU‐80 cells were infected with the concentrated virus at MOI = 1 by using the spin infection method (1500 rpm for 1 h). After 24 h infection, cells were allowed for selection for 2 weeks with 0.2 μg/ml of puromycin. The efficiency of knockdown and overexpression was confirmed by immunoblotting or real‐time PCR method.

### 2.7 Immunofluorescence staining

The method refers to our previous published papers.^18^, ^19^ For detailed information on primary and secondary antibodies, please see Method S1.

### 2.8 BrdU staining assay

For BrdU staining, cells were pre‐grown on coverslips for 24 h and incubated with BrdU (10 μg/ml) for 30 min. After being washed with PBS, the cells were fixed for 20 min in 4% paraformaldehyde. Subsequently, the fixed cells were blocked with 10% goat serum for 1 h and then incubated with a mouse primary monoclonal antibody against BrdU (1:1000, b8434, sigma, Germany) and FITC goat anti‐mouse IgG secondary antibody (Beyotime Biotechnology, China) for 1 h, respectively. DAPI solution (Beyotime Biotechnology, China) was used for nuclear staining and imaged by Leica SP8 LIGHTNING confocal microscopy.

### 2.9 Transwell and wound healing assay

The migration and invasion assays were performed according to our previous studies.^18^, ^19^ Normally, the invasiveness cells were stained 24 h after seeding, whereas when the cells were treated with T5224 inhibitor, the migration and invasion cells were stained 48 h after seeding. In brief, for the scratch wound healing assay, 5 × 10^5^ cells/well were plated into a 6‐well plate and incubated to reach confluence. The monolayer was scratched using a tip, and the cells were then cultured in a complete medium supplemented. The scratched wound was photographed under an Olympus IX71 microscope at 0 h, 12 h and/or 24 h later (details of the steps are in Method S1).

### 2.10 Western blot

Western blot (WB) assay was performed as previously described^18^, ^19^ and detailed in Method S1.

### 2.11 Cell proliferation assay

Cell proliferation was detected by the CCK8 Assay kit (96992, Sigma‐Aldrich, German) following the manufacturer's protocol. In brief, 5 × 10^3^ cells in a 200 μl culture medium were seeded into 96‐well plates. After attachment, 20 μl of CCK‐8 reagents were added to each well every 24 h and then for an additional 4 h incubation, the absorbance was measured at 450 nm on a Tecan Infinite 200 microplate reader (Tecan Group Ltd. Switzerland.)

### 2.12 Spheroid formation assays

Cells (800 cells/mL in a volume of 1 mL) were seeded in Costar® Ultra‐Low Attachment Plates (Corning, USA) and cultured with serum‐free MEM‐EBSS medium containing 1% NEAA, 20 ng/mL of EGF (Invitrogen) and bFGF (Invitrogen) for supplementation. After 7 days of culture, tumour spheres were observed under a microscope.

### 2.13 IHC staining and immune signal evaluation

The immunoreactivity levels of PBRM1 and c‐JUN expression within the cell nucleus of each sample were evaluated under a light microscope by the assessment of the average signal intensity (on a scale of 0‐3). The proportion score of positive cells (0, <5%; 1, 5%‐25%; 2, 26%‐50%; 3, 51%‐75%; 4, 76%‐100%) was independently estimated by two pathologists in the absence of clinical information, as described in our previous studies.^18^, ^19^The intensity and proportion scores were subsequently multiplied to obtain a composite score; the score of 0 to 3 was defined as negative and a score of 4 to 12 as positive (details of the steps are in Method S1).

### 2.14 X‐ray irradiation

A total of 5 × 10^6^ cells were plated into 10 cm cell culture dishes for attachment for 24 h and then irradiated by 6‐MV x‐rays (600 MU/min, Varian Medical Systems). The 0, 2, 4 and 6 Gy of the x‐rays were used to irradiate the cells. After irradiation, the cells were collected for performing cloning formation, Immunofluorescence (IF) or WB assays.

### 2.15 γH2AX foci assay

Immunofluorescence staining of γH2AX was performed according to the IF method described above. The anti‐γH2AX (1:100 dilution, sc‐517348, Santa Cruz, USA) antibody was used for γH2AX foci staining. Cells were then counted by staining with DAPI solution and images were captured by using Leica SP8 LIGHTNING confocal microscopy.

### 2.16 Proteome Profiler Human Phospho‐Kinase Array Kit

The Human Phospho‐MAPK Array Kit (ARY003B, R&D Systems®, Inc. USA) was performed to detect the relative phosphorylation levels of 43 human kinases. Chemiluminescence Gel Imaging System (Bio‐Rad, USA) was used to detect the array's chemiluminescence signal (details of the steps are in Method S1).

### 2.17 RNA isolation and real‐time qPCR

Total RNA was isolated with TRIzol reagent (Invitrogen, USA) and reverse‐transcribed to cDNA with the Prime Script RT Master Mix Kit (TaKaRa). Then the ctDNA served as the template for real‐time PCR.

### 2.18 RNA‐seq and data analysis

Poly(A) mRNA was purified from total RNA (5ug), cleaved into small pieces using divalent cations and reverse‐transcribed to create the final cDNA library with an average insert size of 300 bp (±50 bp). Then the sequencing was performed at the LC‐BIO Technologies (Hangzhou) Co., LTD. The reads were aligned to the homo sapiens reference genome using the HISAT package. The DEGSeq R package (1.18.0)^20^ which refers to the previous research literature was used to determine the differentially expressed genes between groups. The enrichment of differentially expressed genes in KEGG pathways and Gene Ontology (GO) were analysed using KOBAS^16^ software and GOseq R package,^21^ respectively.

### 2.19 ATAC‐seq

The ATAC‐seq was performed at the LC‐BIO Technologies (Hangzhou) according to the standard protocol^22^, ^23^ (details of the protocol are in Method S1).

### 2.20 Statistical analysis

Unpaired two‐tailed student's *t*‐tests and one‐way ANOVA were used to analyse the comparison between two groups and among multiple groups, respectively. The chi‐square test was used for analysing the association between PBRM1 and c‐JUN expression and patient clinicopathological characteristics. Univariate survival and multivariate survival analysis were assessed by the Kaplan–Meier method and a Cox regression model, respectively. All statistical analyses were completed using GraphPad Prism (Version 8.0) and SPSS (Version 13.0).

## 3 RESULTS

### 3.1 Genomic profile of DPC

**FIGURE 1:**
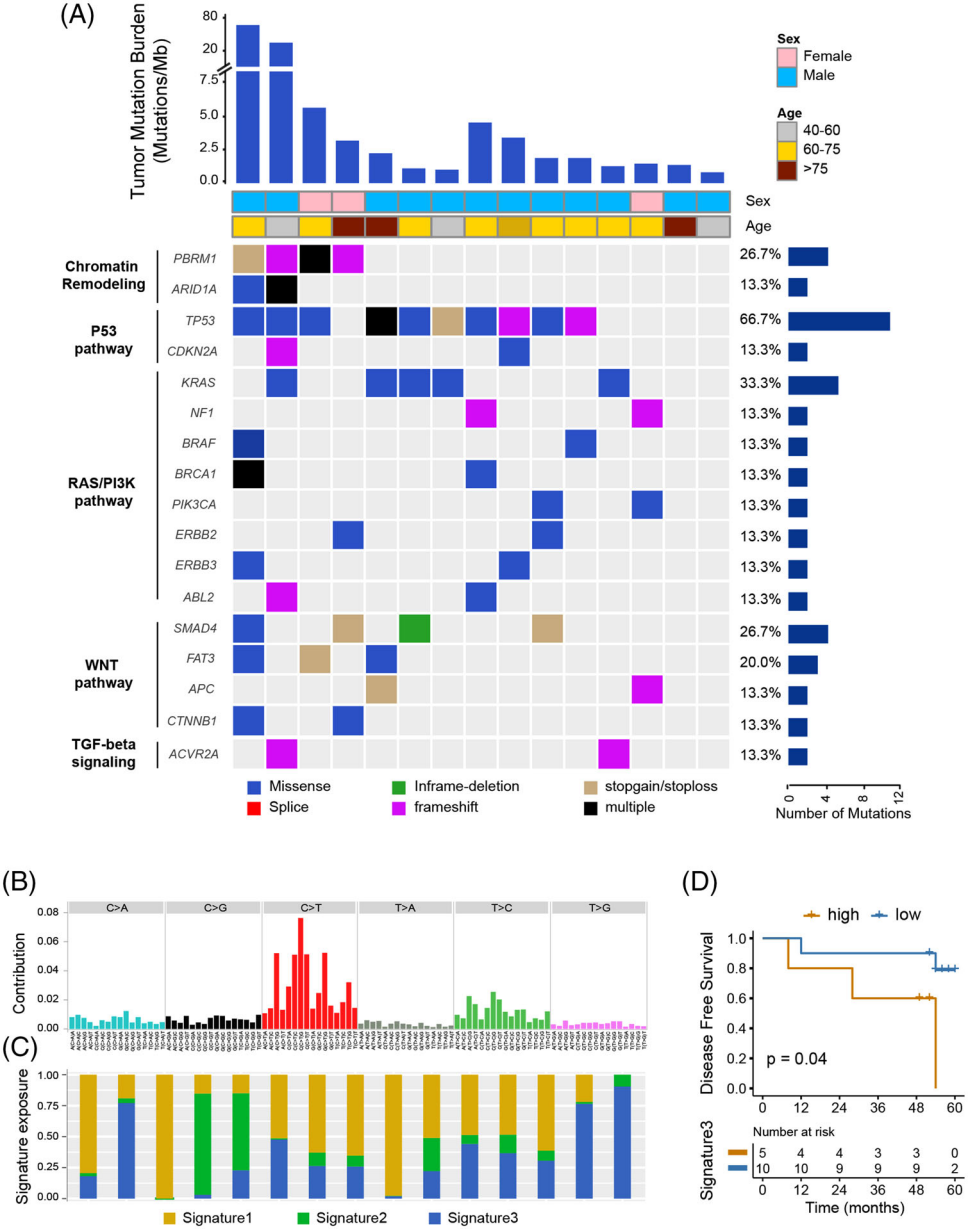
Mutation heatmap and signature in samples of duodenal papillary carcinoma. (A) Selected significantly mutated genes (SMGs) involved in five pathways: chromatin remodelling and the p53, RAS/PI3K‐Akt, WNT and TGF‐beta signalling pathways. (B) Contributions of six possible substitution types in different nucleotide contexts. (C) Heatmap of three mutation signatures from NMF analysis of the mutation spectrum for all samples. (D) Kaplan–Meier curve of survival in this cohort stratified by signature 3 levels (high, yellow line: top 30% in signature 3 exposure; low, blue line: otherwise). NMF, non‐negative matrix factorisation.

To understand the genomic landscape of DPC, we performed whole‐exome sequencing of tumour tissues located in duodenal papilla from 15 patients, along with sequencing of matched normal tissues (tumour, 167×; normal, 164×). In total, 3951 nonsynonymous somatic SNVs were identified, with a median of 263 mutations (range, 25 to 2 344) in each sample. The tumour mutation burden (TMB) for each patient was calculated, and the median TMB was 1.9/Mb (range, 0.82 to 77.1 mutations/Mb). The top mutated known cancer genes were *TP53* (66.7%), *KRAS* (33.3%), *SMAD4* (26.7%), *PBRM1* (26.7%), *FAT3* (20.0%), *CDKN2A* (13.3%) and *ARID1A* (13.3%). Notably, except for the *PBRM1* gene, all other genes were previously reported in other ampullary carcinoma cohorts. The significantly mutated genes were part of five pathways: RAS/PI3K, P53, WNT, TGF‐β and chromatin remodelling pathways (Figure 1A).

Among the six possible single‐base substitutions, the most dominant base substitution was C > T transitions, 42% of which were CpG to TpG (Figure 1B). Nonnegative matrix factorization was used to evaluate the mutation signatures associated with DPC, and we identified three prominent signatures (Figure 1C). Signature 1 is highly consistent with COSMIC signature 1 (cosine similarity = 0.93), which is common among all cancers and proposed to be driven by the process of spontaneous deamination of 5‐methylcytosine. Signatures 2 and 3 did not correspond to any of the reported COSMIC signatures. These results were consistent with previous observations suggesting that no special environmental exposure drives DPC development. In addition, we found that signature 3 was associated with poor outcomes in our study set (log‐rank test, *p* = .04) (Figure 1D).

### 3.2 Major altered pathways and PBRM1 mutations in DPC

**FIGURE 2:**
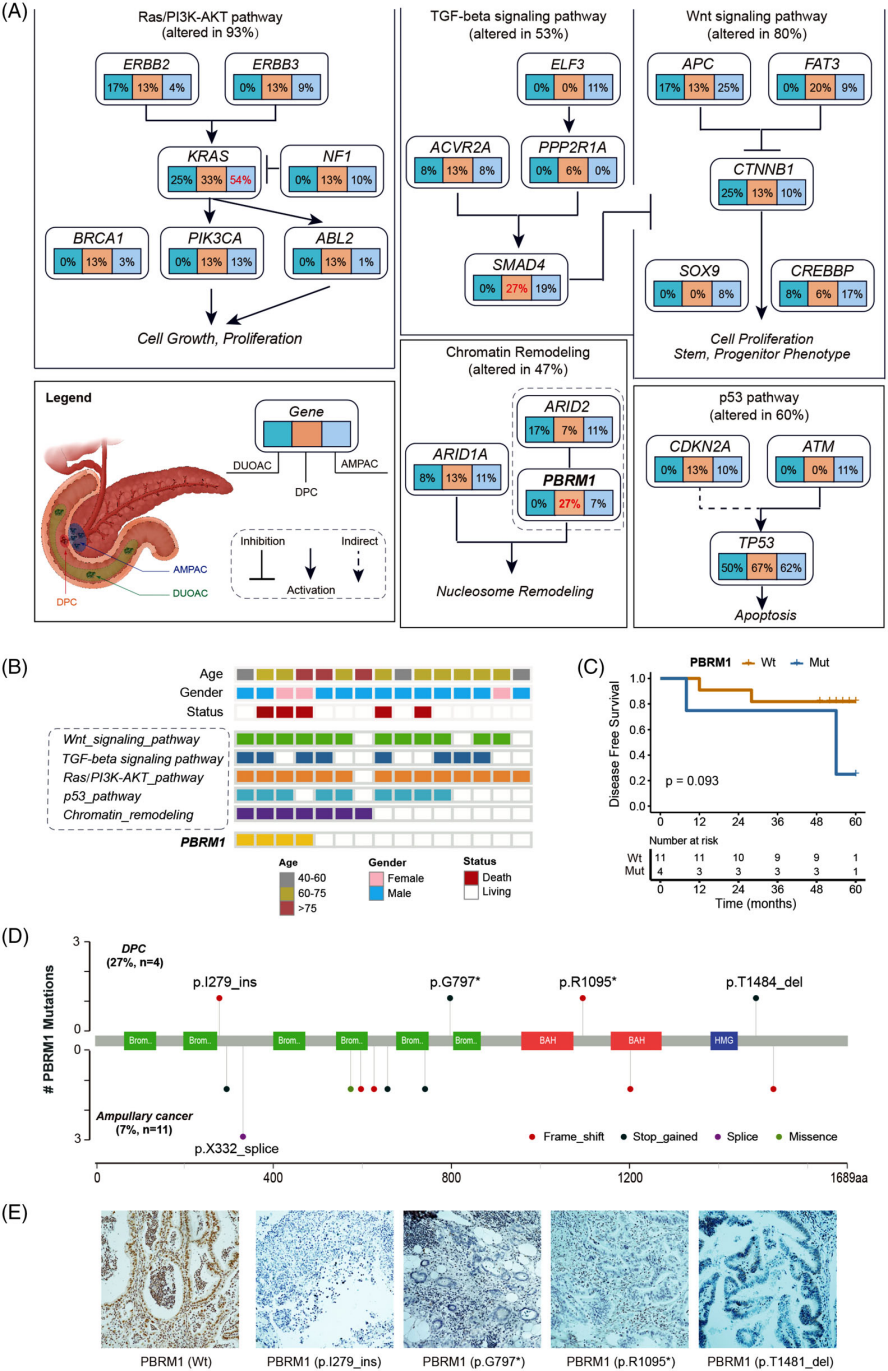
Major altered pathways and PBRM1 mutations involved in chromatin remodelling. (A) Mutation frequency of major mutated pathways defined by somatic mutations are expressed as a percentage of cases for each gene in duodenal adenocarcinoma (DUOAC, the green box), duodenal papillary carcinoma (DPC, the earth yellow box) and ampullary adenocarcinoma (AMPAC, the light blue box). Legend marks the anatomical structure of the three tumours. (B) Genetic alterations in mutated genes grouped by pathway are illustrated for each patient. Note: PBRM1 was mutated in 27% (4/15) of cases. (C) The association of PBRM1 mutation and survival approached significance in this small cohort. (D) Mutation map of PBRM1 genes in DPC and AMPAC, in which each lollipop denotes a unique mutation location. (E) Immunohistochemistry of PBRM1 in one wild‐type and four mutant samples.

We then combined the somatic mutations at the gene level within the five pathways (RAS/PI3K, P53, WNT, TGF‐β and chromatin remodelling) to assess the impact of these pathways among the three anatomical sites: DUOAC, DPC and AMPAC. The mutation frequencies in altered genes per tumour type are illustrated in Figure 2A. Notably, *PBRM1*, a known tumour suppressor gene in renal clear cell carcinoma,^24^, ^25^, ^26^ was recurrently mutated in 27% (4/15) of our DPC cohort, which was significantly higher than that in the DUOAC (0%, *p* < .001) and AMPAC (7%, *p* < .001) cohorts. RAS/PI3K (93%, 14/15) was the most frequently mutated pathway in our DPC cohort, followed by Wnt signalling (80%, 12/15), P53 (60%, 9/15), TGF‐β signalling (53%, 8/15) and the chromatin remodelling pathway (47%, 7/15) (Figure 2B). As a result, it was found that three of four patients (75%) with *PBRM1* mutations experienced tumour progression and died. We then performed a survival analysis of the *PBRM1*‐mutant versus *PBRM1*‐wt groups. Patients with tumours harbouring *PBRM1* mutations appeared to have a shorter DFS, although this was not significant (*p* = .093) in this small cohort (Figure 2C). Upon further analysis, we found that all four *PBRM1* mutations were loss‐of‐function (LOF) mutations and were different from those previously reported in AMPAC (Figure 2D). To further validate whether loss‐of‐function mutation of *PBRM1* affected protein expression, we performed IHC approaches to determine the expression of PBRM1 protein in four patients harbouring PBRM1 mutations and one wild‐type patient. The results confirmed that the expression of PBRM1 protein was lost in all four cases with an inactivating mutation, whereas the wild‐type cases retained expression (Figure 2E).

### 3.3 Knockdown of PBRM1 promotes cancer cell progression and epithelial–mesenchymal transformation

**FIGURE 3:**
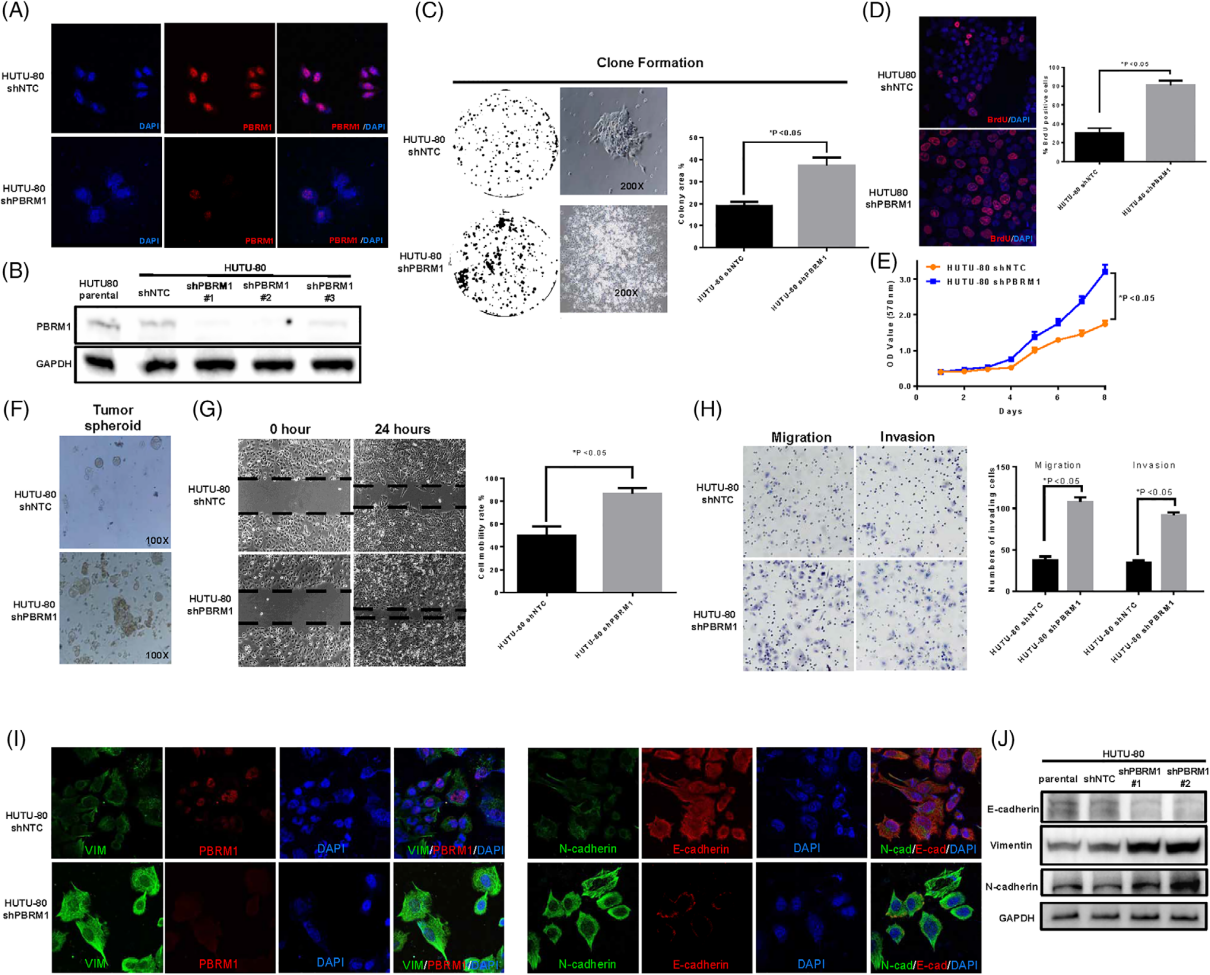
Knockdown of PBRM1 promotes HUTU‐80 cell progression and epithelial–mesenchymal transformation. (A) IF result of PBRM1 expression in HUTU‐80‐shNTC and HUTU‐80‐shPBRM1 cells. (B) WB results of PBRM1 expression in HUTU‐80‐shNTC and HUTU‐80‐shPBRM1 cells. (C) Downregulation of PBRM1 expression resulted in an increase in colony formation in HUTU‐80‐shPBRM1 cells. (D) Downregulation of PBRM1 expression resulted in an increase in proliferation in HUTU‐80 cells (BrdU staining). (E) Downregulation of PBRM1 expression resulted in an increase in proliferation in HUTU‐80 cells (MT method). (F) Downregulation of PBRM1 expression resulted in an increase in tumour spheroid formation ability in HUTU‐80‐shPBRM1 cells. (G) Wound healing result. (H) Downregulation of PBRM1 expression resulted in increases in the migration and invasion abilities in HUTU‐80 cells. (I) IF results of VIM, PBRM1, N‐cadherin and E‐cadherin expression in HUTU‐80‐shNTC and HUTU‐80‐shPBRM1 cells. (J) WB analysis of VIM, N‐cadherin and E‐cadherin expression in HUTU‐80‐shNTC and HUTU‐80‐shPBRM1 cells. (*p* < .05). Note: Experiments were performed in triplicate and repeated three times with similar results. Bars display mean ± S.D.

Given that all four *PBRM1* mutations were loss‐of‐function mutations and PBRM1 has been reported in other cancer types (renal clear cell carcinoma) as a tumour suppressor,^24^, ^25^, ^26^, ^27^ we set out to understand the functions and potential mechanisms of PBRM1 alteration using shRNA targeted against *PBRM1* in the HUTU‐80 cell line (the only cancer cell line of DUOAC) (Figure 3A,B). Downregulation of PBRM1 expression resulted in increased colony formation, proliferation, wound healing, migration and invasion in HUTU‐80 cells (Figure 3C–H, and Figure S2A–D). At the same time, overexpression of PBRM1 in HUTU‐80 cells inhibited the proliferation, migration, colony formation and invasion abilities of HUTU‐80 cells (Figure S1A–E). In addition, MDA‐MB‐231 breast cancer cells (PBRM1 I228V missense mutation) were also used to rescue PBRM1 expression and to validate the functions of PBRM1 and obtained similar results. At the same time, we observed a mesenchymal transformation phenotype in HUTU‐80 cells after knockdown of PBRM1 expression, mainly manifested as decreased protein levels of E‐cadherin and increased expression of vimentin (VIM) and N‐cadherin (Figure 3I,J and Figure S2E,F). The short‐term experiments were repeated with shRNA #2 with similar results. These observed phenotypic changes reflected that silencing PBRM1 could promote cancer cell progression and epithelial‐to‐mesenchymal transition (EMT).

### 3.4 Dysregulated transcription factors after silencing PBRM1 in HUTU‐80 cells

To identify genes regulated by PBRM1, we performed RNA‐seq of PBRM1 knockdown HUTU‐80 cells (shPBRM1) and control (shNTC) (*n* = 2 biological replicates per group). After *PBRM1* knocking down, 2212 genes were upregulated and 1308 genes were downregulated (fold change = 1.5, *q* value < 0.01) (Figure S3). Given the role of *PBRM1* as a chromatin remodeller, we further analysed the RNA‐seq data to explore the effect on transcription factor (TF) expression. After further filtering by the human transcription factor database JASPAR (https://jaspar.genereg.net/), we found that a total of 149 transcription factors (79 were increased and 70 were decreased) were significantly changed after PBRM1 knockdown.

### 3.5 Silencing of PBRM1 leads to dysregulation of transcription factors involved in chromatin remodelling

**FIGURE 4:**
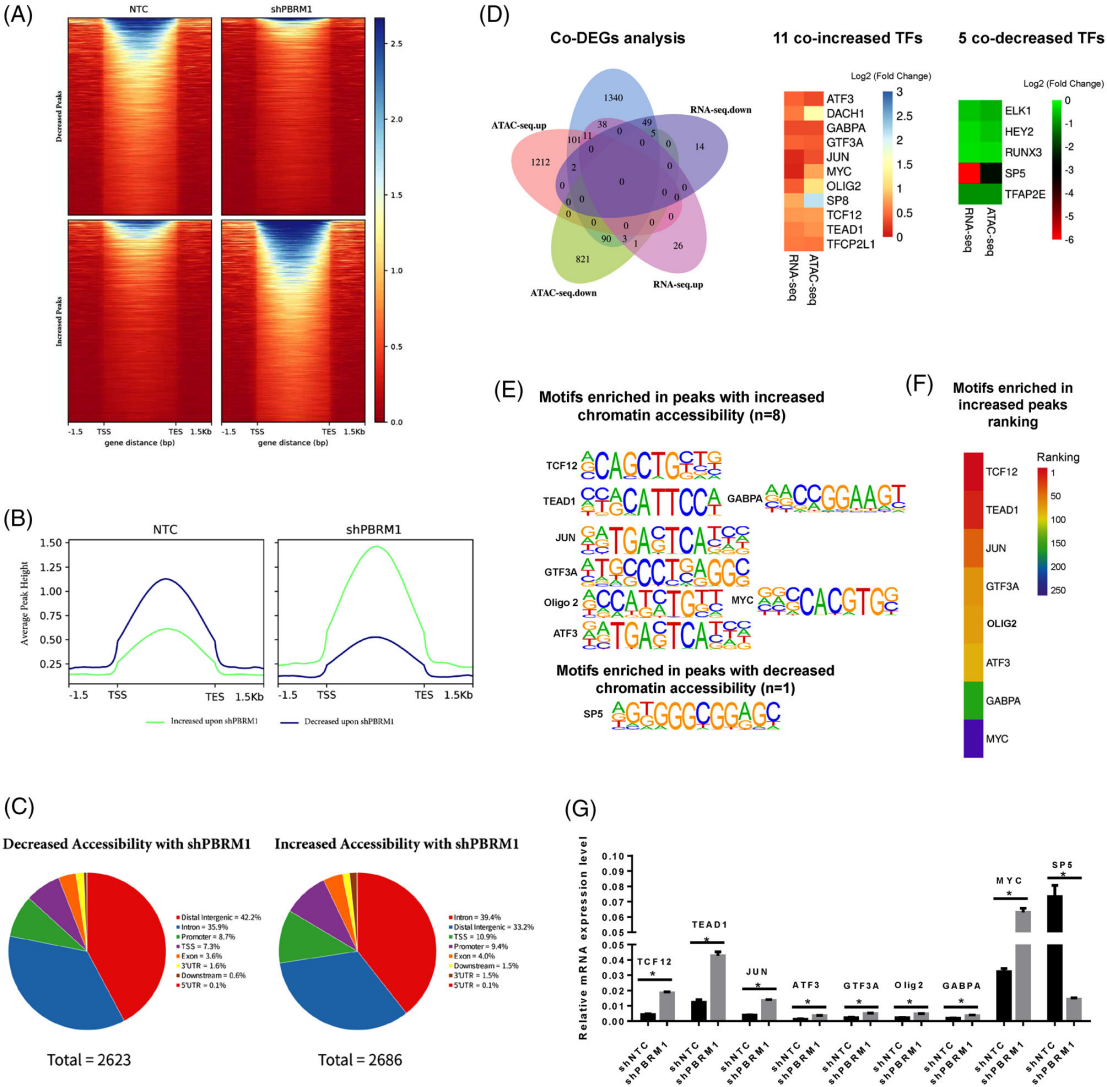
The combined analysis of the differential chromatin accessibility genes by ATAC‐seq and differentially expressed TFs by RNAseq. (A) Regions of differential chromatin accessibility with at least 1.5‐fold. (B) Metagene plots of the regions identified as differential chromatin accessibility with PBRM1 knockdown using ATAC‐seq. (C) The proportion of genomic elements associated with the increased or decreased chromatin accessibility peaks. (D) Venn diagram analysis of the differential chromatin accessibility genes by ATAC‐seq and differentially expressed genes by RNAseq. 11 co‐increased and five co‐decreased TFs were obtained. (E) MOTIF analysis was performed of the 16 TFs using HOMER, and nine (8 increased and 1 decreased) of them were found to have enriched motifs in peaks. (F) Among the eight increased TFs, the top three are TCF12, TEAD1 and JUN. (G) Transcript level changes of nine differentially expressed TFs were validated by Qrt‐PCR (*p* < .05). TF, transcription factor.

To further identify whether these 149 significantly changed TFs were directly regulated by PBRM1's chromatin remodelling function, we used the ATAC‐seq to identify chromatin accessibility changes and PBRM1‐dependent TF sites after PBRM1 knockdown. Although PBRM1 knockdown did not have a dramatic effect on global chromatin accessibility, there was a significant decrease in chromatin accessibility at 2623 peak sites and increased accessibility at 2686 peaks in HUTU80‐shPBRM1 cells with similar genomic distributions (Figure 4A–C). Among these peak sites, a total of 2246 different peak sites were TF binding sites, 1326 genes had an increase in chromatin accessibility and 920 genes had decreased chromatin accessibility based on ATAC‐seq data with the selection criteria of |log2(shPBRM1/NTC)| > 0.3 and *p* < .001. Then, Venn diagram analysis was used to further analyse whether these differentially increased and decreased chromosome accessibility genes were correlated with transcription level changes based on the RNA‐seq data. The results showed that there were 11 co‐increased and 5 co‐decreased genes between chromatin accessibility and the transcription level data upon shPBRM1 transfection, respectively (Figure 4D). MOTIF analysis was then performed of the 16 TFs using HOMER, and 9 (8 increased and 1 decreased) of them were found to have enriched motifs in peaks. Among eight increased TFs, the top three were TCF12, TEAD1 and JUN (Figure 4E,F) and these results were also validated using real‐time PCR (qRT–PCR) (Figure 4G).

### 3.6 Silencing of PBRM1 promotes HUTU‐80 EMT through the c‐JUN/VIM axis

**FIGURE 5:**
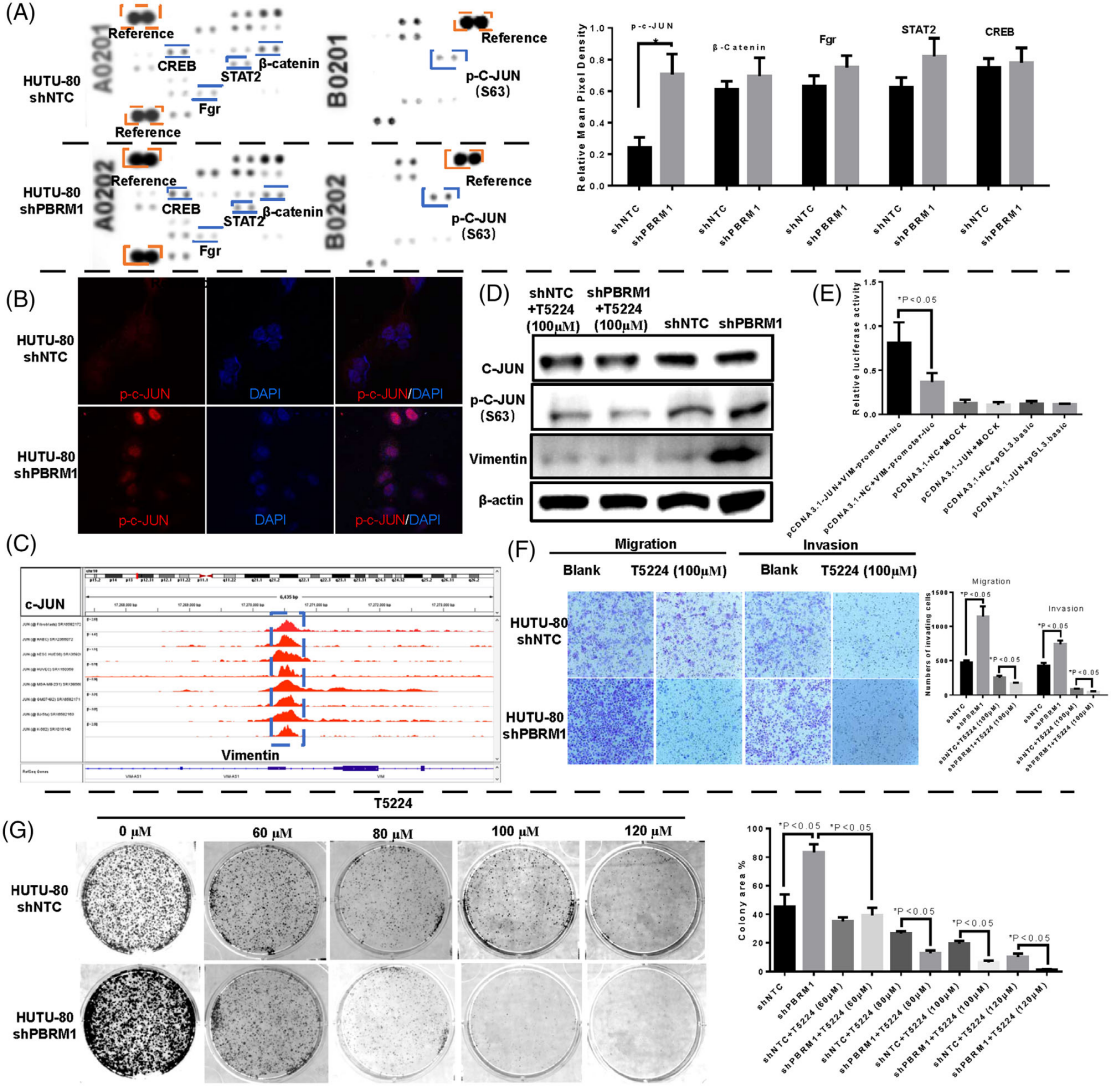
Downregulation of PBRM1 promotes HUTU‐80 invasiveness through the c‐JUN/VIM axis. (A) The phosphorylation profiles of kinases upon shPBRM1 transfection in HUTU‐80 cells were detected with a Proteome Profiler Human Phospho‐Kinase Array Kit. (B) The nuclear location of p‐c‐JUN was increased in HUTU‐80 shPBRM1 cells. (C) ChIP data of the c‐JUN‐binding region in the VIM promoter region from the ChIP‐Atlas database. (D) The expression levels of p‐c‐JUN and vimentin were increased upon PBRM1 knockdown and could be inhibited by treatment with 100 μM T5224. (E) The dual‐luciferase assay reported that c‐JUN directed binding in the vimentin promoter region. (F) The c‐JUN inhibitor (T5224) significantly inhibited the invasion promotion effect of PBRM1 knockdown, and PBRM1 knockdown cells were much more sensitive to the c‐JUN inhibitor T5224 than shNTC cells. (G) PBRM1 knockdown cells were much more sensitive to different concentrations of T5224 than shNTCs. (*p* < .05). Note: Experiments were performed in triplicate and repeated three times with similar results. Bars display mean ± S.D.

We further sought to understand changes in transcription factors and the phosphorylation profiles of kinases upon shPBRM1 transfection in HUTU‐80 cells. We found that the expression of p‐c‐JUN was increased in HUTU‐80‐shPBRM1 cells (Figure 5A), and this increase in c‐JUN was reflected in the ATAC‐seq and RNA‐seq results above. At the same time, an increased immunofluorescence signal of c‐JUN in the nucleus was observed after PBRM1 knockdown, indicating that c‐JUN is activated after PBRM1 knockdown (Figure 5B). Therefore, we focused on c‐JUN as a target gene after the downregulation of PBRM1.

c‐JUN, as known as AP‐1, is an onco‐transcription factor that has been linked to providing signals for cell survival, cell motility and invasiveness which is highly overexpressed in many human cancer types.^28^, ^29^ Since we observed that knockdown of PBRM1 expression in HUTU‐80 cells formed an EMT phenotype and increased c‐JUN expression, we inferred that the activation of c‐JUN might involve in the EMT changes. Then, we found that there were binding sites of c‐JUN on the promoter regions of two EMT‐regulating genes (*VIM* and *N‐cadherin*), which were predicted by Promo software and the JASPAR database. We cloned the upstream promoter regions of VIM and N‐cadherin into a pGL3‐basic luciferase reporter plasmid, and a dual‐luciferase assay showed that the activity of VIM was significantly increased in c‐JUN‐overexpressing cells compared with control vector cells (Figure 5E), whereas there was no significant change in N‐cadherin group (data not shown). Consistent with this result, the chromatin immunoprecipitation (ChIP) data from the ChIP‐Atlas database (http://chip‐atlas.org/) showed that there was a strong c‐JUN‐binding region in the VIM promoter region (Figure 5C). Furthermore, the in vitro invasion experiment results indicated that T‐5224, a small molecule c‐JUN inhibitor that specifically inhibits the binding of c‐JUN to the promoter region of its target genes, could significantly reverse the invasion promotion effect of PBRM1 knockdown compared to shNTC cells (Figure 5D,F,G). Therefore, we speculate that c‐JUN plays a role in EMT and invasion promotion after PBRM1 knockdown by upregulating VIM expression.

### 3.7 PBRM1 expression was negatively associated with c‐JUN expression and predicted poor prognosis

Next, a validation cohort of DPC patients (*n* = 116 cases of DPC) was selected for PBRM1 and c‐JUN IHC staining, and representative IHC images are shown in Figure S4. The PBRM1 positive expression rate was significantly decreased from 48.8% (42/86) to 26.7% (8/30) in the patients without nerve invasion to those with nerve invasion (*p* < .05, Table 1). Furthermore, no other significant correlations were found between PBRM1 expression and clinicopathological factors, such as sex, differentiation, size, TNM grade, lymphatic invasion and vascular invasion, as shown in Table S1.

At the same time, the patients with positive expression of c‐JUN had larger tumour sizes, deeper invasion depths and more aggressive nerve invasion and vascular invasion than the negative patients (*p* < .05, Table 1). Furthermore, PBRM1 expression was found to be negatively correlated with c‐JUN expression in PDC patients (Pearson correlation = −0.527, *p* < .05). In the present cohort of PDC patients, 97 patients had a 5‐year follow‐up, and the Cox multivariate analysis indicated that only vascular invasion was an independent prognostic factor of this cohort of DPCs (*p* = .009, HR = 6.850), whereas PBRM1 (*p* = .505, HR = 0.444) and c‐JUN (*p* = .466, HR = 0.531) expression status were not independent prognostic factors.

**FIGURE 6:**
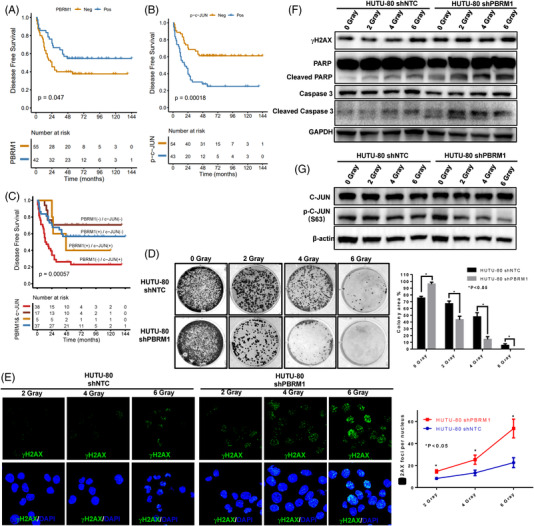
Loss of PBRM1 expression predicted poor prognosis in DPC and sensitization to irradiation treatment. (A) Survival curve of patients with negative or positive PBRM1 expression in DPC. (B) Survival curve of patients with negative or positive c‐JUN expression in DPC. (C) Survival curve of the correlation between PBRM1 and c‐JUN expression in DPC. (D) HUTU‐80‐shPBRM1 cells resulted in fewer colonies than HUTU‐80‐shNTC cells after irradiation at 2, 4 and 6 grays. (E) HUTU‐80‐shPBRM1 cells had a higher number of γh2AX foci than HUTU‐80‐shNTCs after irradiation. (F) WB analysis of γH2AX, cleaved caspase‐3 and cleaved PARP. (G) Western blot analysis of the expression changes of c‐JUN and p‐c‐JUN in response to irradiation. (*p < .05). Note: Experiments were performed in triplicate and repeated three times with similar results. Bars display mean ± S.D. DPC, duodenal papillary carcinoma; WB, Western blot.

Furthermore, the association between PBRM1 and c‐JUN status and the prognosis of PDC was analysed. The 3‐ and 5‐year overall survival rates of the patients were 50.5% and 45.1%, respectively. The survival of patients with PBRM1‐positive was significantly longer than that of PBRM1‐negative patients (52.0 months vs. 24.0 months, *p* = .047, Figure 6A). At the same time, the survival time in c‐JUN‐positive patients was significantly shorter than that in c‐JUN‐negative patients (54.5 months vs. 19.0 months, *p* < .005, Figure 6B). Moreover, these DPC patients could be divided into four subgroups when a joint analysis of c‐JUN and PBRM1 expression was performed (Figure 6C). The patients with both negatives (PBRM1−/c‐JUN−) had a much better prognosis (the 5‐year survival rate was 70.6%) than single‐positive or double‐positive cases. The patients with PBRM1−/c‐JUN+ had the worst prognosis (the 5‐year survival rate was 23.0%), whereas the PBRM1+/c‐JUN− or PBRM1+/c‐JUN+ patients had a moderate prognosis (the 5‐year survival rates were 56.8% and 40.0%, respectively, Figure 6C, *X* ^2^ = 17.447, *p* = .001).

### 3.8 Silencing of PBRM1 expression in HUTU‐80 cells was sensitive to radiation

**FIGURE 7:**
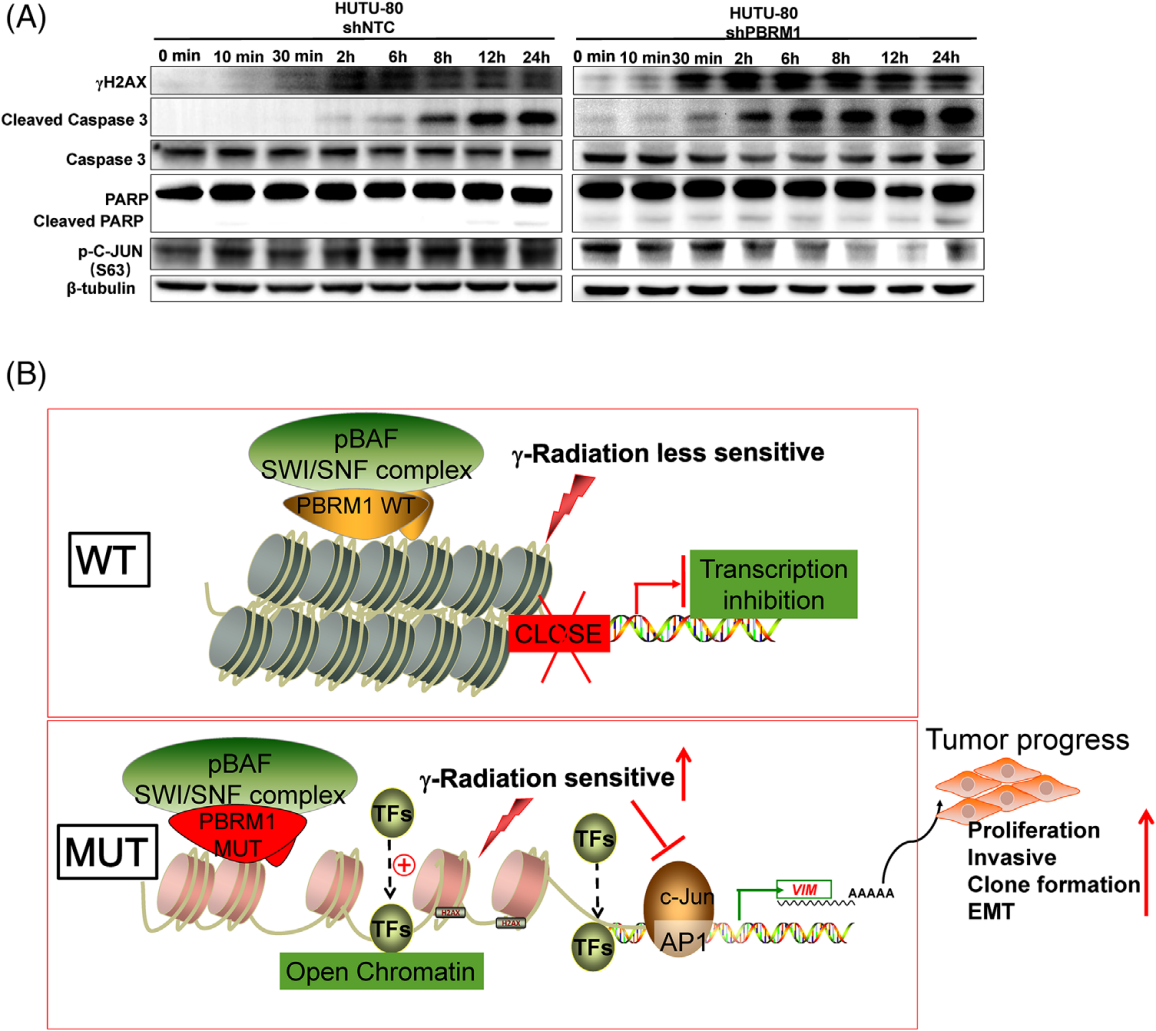
Time dynamic observation of irradiation treatment and the proposed model for the mechanism of PBRM1 in DPC. (A) Time dynamic observation of the expression of γH2AX, caspase‐3, c‐JUN and PARP in HUTU‐80‐shPBRM1 and HUTU‐80‐shNTC cells after irradiation. (B) The proposed model for the mechanism of PBRM1 in DPC. DPC, duodenal papillary carcinoma.

In recent years, radiotherapy has been increasingly used in a variety of cancer treatments, including CRC. Considering that low expression of PBRM1 in DPC patients predicted a poor prognosis, we continue to examine the influence of PBRM1 silencing on cellular radiosensitivity. After irradiation at 2, 4 and 6 grays, HUTU‐80‐shPBRM1 cells resulted in fewer colonies than HUTU‐80‐shNTC cells (Figure 6D). PBRM1 knockdown cells had a higher number of γH2AX foci, a hallmark of DNA damage, than HUTU‐80‐shNTC cells, suggesting that more DNA double‐strand breaks were induced by irradiation (Figure 6E). Western blot analysis showed dramatic increases in Γh2AX, cleaved caspase‐3 and cleaved PARP in HUTU‐80‐shPBRM1 cells compared with control HUTU‐80‐shNTC cells after irradiation (Figure 6F). These data suggested that silencing PBRM1 promotes irradiation‐induced cell death. Our above studies have shown that c‐JUN activation was involved in the tumour‐promoting effect of PBRM1 in HUTU‐80 cells. Therefore, we also tested whether the increased sensitivity of HUTU‐80‐shPBRM1 cells to radiation was due to the inhibition of c‐JUN activation by radiation. More interestingly, we found that p‐c‐JUN expression was significantly inhibited in HUTU‐80‐shPBRM1 cells after irradiation, whereas there were no visible significant changes in HUTU‐80‐shNTC cells (Figure 6G). Moreover, we found that p‐c‐JUN expression began to be significantly decreased in HUTU‐80‐shPBRM1 cells at 6 h after four grays of irradiation, whereas there was no significant change in HUTU‐80‐shNTC cells (Figure 7A). At the same time, the expression levels of γh2AX, cleaved caspase‐3 and cleaved PARP were also significantly increased in HUTU‐80‐shPBRM1 cells compared to HUTU‐80‐shNTC cells in a time‐dependent manner (Figure 7B). Thus, PBRM1 downregulation sensitizes cells to irradiation, which may be due to the suppression of c‐JUN by irradiation.

## 4 DISCUSSION

DPC is a rare malignant cancer occurring at the location of the duodenum. Due to its rarity, DPC is seldom studied as a unique disease, and no specific treatment guideline is provided. DPC is often compared with CRC or gastric cancers for treatment plans, and the outcome of DPCs is still less than ideal.^2^ In this study, we performed whole‐exome sequencing of 15 cases of DPC to better understand the molecular pathogenesis of this tumour type.

Our results confirmed that DPC shares some common molecular features with CRC, AMPAC and DUOAC, with similar TP53, TGF‐β, PI3K/Akt and Wnt pathways.^7^, ^8^, ^9^ Several potentially targetable gene mutations were also identified in our DPC cohort, including *PIK3CA* and *ERBB2* alterations. Thus, the molecular data of DPC suggest that clinical testing for these signalling statuses might be beneficial to patients, which could soon become a stepping stone to personalized medicine.

Furthermore, we also observed some mutation patterns different from those in CRC, AMPAC and DUOAC. Unlike a previous study that indicated that AMPAC harbour an *ELF3* tumour suppressor gene mutation, we did not find an *ELF3* mutation in DPC.^8^ Instead, we identified frequent alterations in the *PBRM1* gene involved in the chromatin remodelling pathway. In our present cohort of DPC, seven out of 15 cases achieved chromatin remodelling pathway dysregulation, and among them, PBRM1 mutation accounted for the largest proportion, nearly three times higher than AMPAC.^8^ Previous studies have reported that chromatin remodelling *SWI/SNF* subunit genes are mutated in the top rankings in many human tumours.^30^, ^31^ Recent examples include *ARID1A* in ovarian clear cell carcinoma and transitional bladder cancer,^32^, ^33^ *PBRM1* in renal cell cancers^34^ and *ARID2* in hepatocellular carcinoma (HCC) and melanoma and act as a cancer suppressor function in cancers.^35^ Here, we first reported that *PBRM1* was the most frequently inactivated gene among the dysregulated chromatin remodelling pathway genes in DPC. We also infer that chromatin remodelling pathway dysregulation may play an important role in DPC initiation and progression. The *PBRM1* gene encodes a subunit of the *SWI/SNF* chromatin‐remodelling complex and forms a complex with *ARID2*. *ARID2* partial or complete loss of physiological function is present in many cancers and is thought to play vital roles in carcinogenesis and cancer progression.^36^ Similarly, in this study, in vitro cell experiments showed that downregulation of PBRM1 expression could significantly promote the proliferation and migration of duodenal tumour cells and play an important regulatory role in EMT. At the same time, the clinical DPC tissue IHC data further confirmed that the patients who lost PBRM1 expression had a poor prognosis.

The SWI/SNF multiprotein complex was reported to serve as an important determinant of genomic plasticity, which regulates the accessibility of TFs to DNA and influences a variety of biological processes, including cancer cell invasion and cell proliferation. To support this inference, from our RNA‐seq and ATAC‐seq data, downregulation of PBRM1 could modulate the accessibility of many TFs to DNA. C‐JUN was one of the transcription factors with increased accessibility based on PBRM1 knockdown.

c‐JUN, known as a component of AP‐1, was first identified as a viral oncoprotein and is frequently activated in human cancers.^29^, ^37^ Many stimulatory signals, including oncoproteins and growth factors, activate c‐JUN‐dependent transcription and play important roles in carcinogenesis and cancer progression.^38^ Our data show that knockdown of PBRM1 not only enhanced the chromatin accessibility and transcription of c‐JUN but also enhanced the phosphorylation and activation of c‐JUN at serine 63. Additionally, activated c‐JUN promotes the epithelial–mesenchymal transformation of duodenal tumour cells by upregulating the expression of VIM to enhance their invasion and migration abilities, resulting in tumour progression. Furthermore, the IHC data also confirmed that high c‐JUN expression was negatively correlated with PBRM1 and predicted a poor prognosis in PDC. Combined with the expression of PBRM1 and c‐JUN in DPC, it was found that patients with both low PBRM1 and high c‐JUN expression had a worse prognosis than other single‐positive or double‐negative patients.

DPCs are often classified as CRC in treatment options. However, our molecular characterization indicated that they are a group of cancers with heterogeneous profiles that are different in many ways from CRC. Given our findings, DPCs may need to be treated differently based on their molecular markers, and the PBRM1‐c‐JUN‐VIM axis might serve as a valid target, particularly a subgroup of DPCs with loss of PBRM1.

Radiotherapy is a remarkably effective cancer treatment strategy, with nearly half of all patients receiving radiotherapy as a curative or palliative treatment.^39^ In response to radiation, DNA is damaged directly or indirectly through DNA breaks or replication stress and a DNA damage response is triggered. A majority of DNA lesions directly caused by irradiation are cyclobutene pyrimidine dimers (CPDs), which are primarily repaired by nucleotide excision (NER). Dysregulation of NER mechanisms plays a crucial role in carcinogenesis.^40^ Previous reports have confirmed that ARID2 mutation or deficiency results in an impairment of NER by abrogating the recruitment of XPG to UV irradiation DNA damage sites and contributing to sensitivity to UV irradiation.^40^ A previous study demonstrated that ARID2 deficiency could enhance the sensitivity of cells to DNA‐damaging agents.^41^ Moreover, a cooperative manner of frequent joint inactivation of ARID2, PBRM1 and BAP1 in the subtype of hepatic carcinomas may contribute to malignancy.^42^ Thus, given that it forms a PBAF complex with *ARID2*, we hypothesized that *PBRM1* may also be involved in the DNA damage response. Here, we found that silencing PBRM1 sensitized cells to irradiation‐induced cell arrest, reflected by a smaller number of colonies and dramatic increases in γh2AX, cleaved caspase‐3 and cleaved PARP expression. Therefore, these approaches warrant further exploring as possible treatments for PBRM1 mutated DPC.

In summary, we described the mutational landscape of DPC for the first time and found that it was a group of rare tumours with unique molecular characteristics and some shared characteristics with DUOAC and AMPAC. A high frequency of dysregulation in the chromatin remodelling pathway, especially PBRM1‐inactivating mutations and the PBRM1‐c‐JUN‐VIM axis, served as an important role of cancer invasion and was associated with poor patient outcomes in DPC. Moreover, the PBRM1‐deficient cell lines were shown more sensitive to irradiation, and targeted inhibition of c‐JUN can prevent tumour progression caused by PBRM1 deficiency. Thus, PRBM1 is involved in the development of DPC, but its exact function during tumour development needs to be further investigated.

## 5 CONCLUSION

In this study, we described the mutational landscape and the unique molecular characteristics of DPC, especially PBRM1‐inactivating mutations and the PBRM1‐c‐JUN‐VIM axis, which serve as important methods of cancer invasion and were associated with poor patient outcomes in DPC. Radiotherapy or targeting c‐JUN may also be a potential treatment strategy for patients with PBRM1 deficiency. Our study expands the understanding of the molecular characteristics of DPC and suggests PBRM1 as a potential therapeutic target and prognosis indicator for DPC.

## CONFLICT OF INTEREST

Honglin Zhu and Tonghui Ma are the employees of Genetron Health (Beijing) Technology, Co. Ltd., Beijing, China. The other authors declare no conflict of interest for this article.

## Supporting information

Supporting Information

Supporting Information

Supporting Information

Supporting Information

Supporting Information

Supporting Information

Supporting Information

Supporting Information
