# Supplementary figures and images for: PBRM1 presents a potential prognostic marker and therapeutic target in duodenal papillary carcinoma
Source: Clin Transl Med. 2022 Sep 30;12(10):e1062. doi: 10.1002/ctm2.1062 (PMC9523678; doi:10.1002/ctm2.1062)

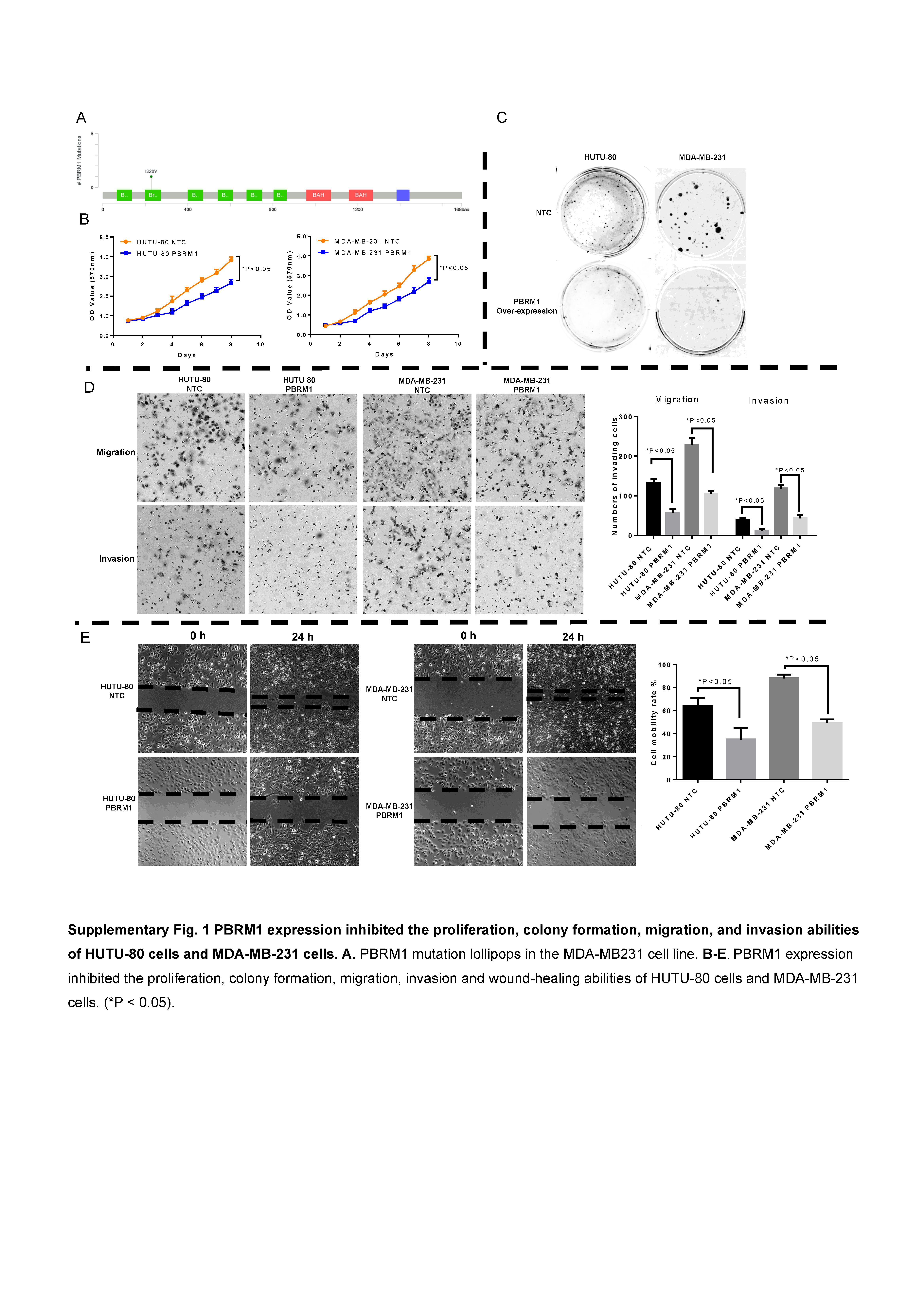

Supplement: Supplementary file 1 — Supporting Information [file CTM2-12-e1062-s008.tiff]

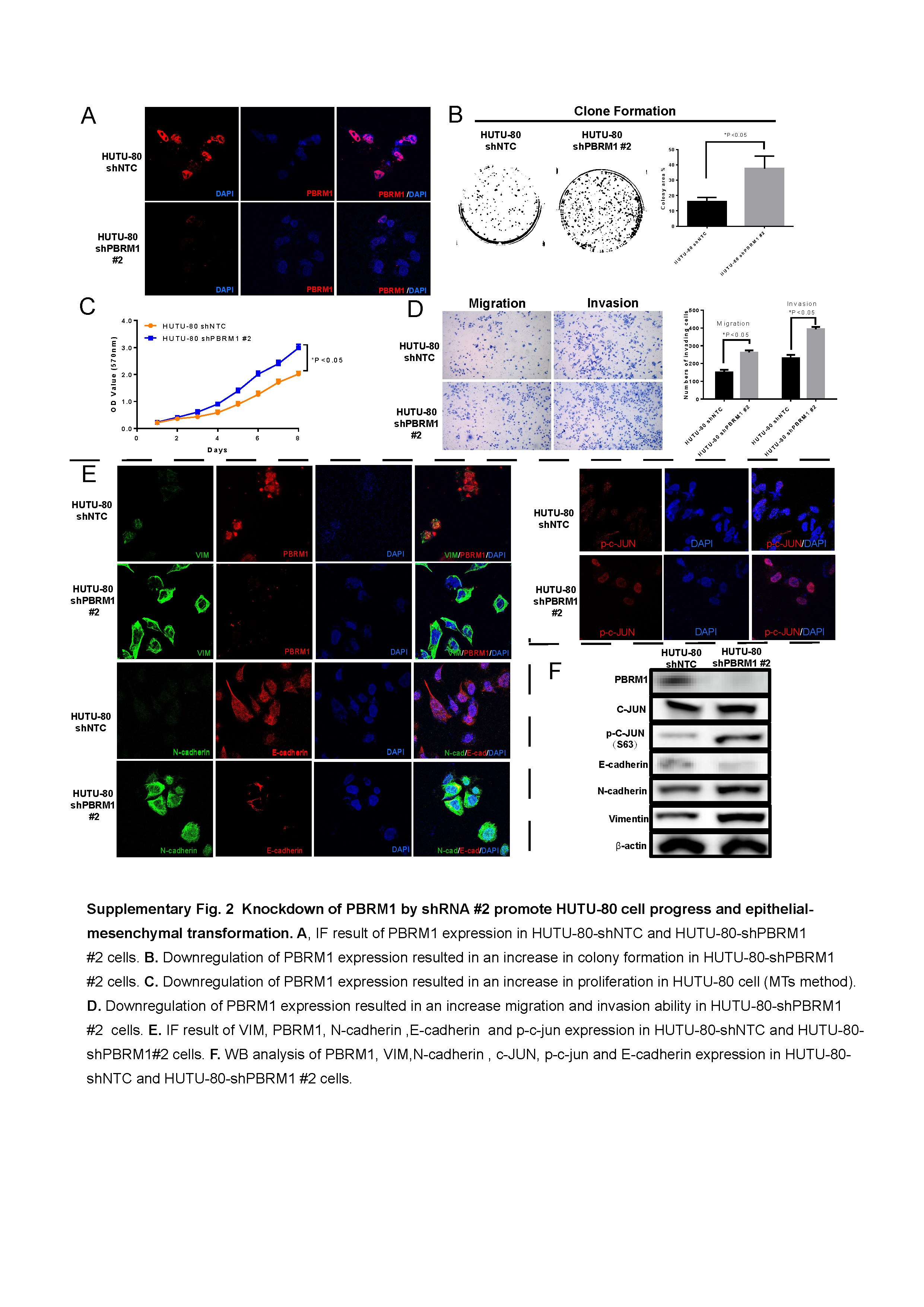

Supplement: Supplementary file 2 — Supporting Information [file CTM2-12-e1062-s006.tiff]

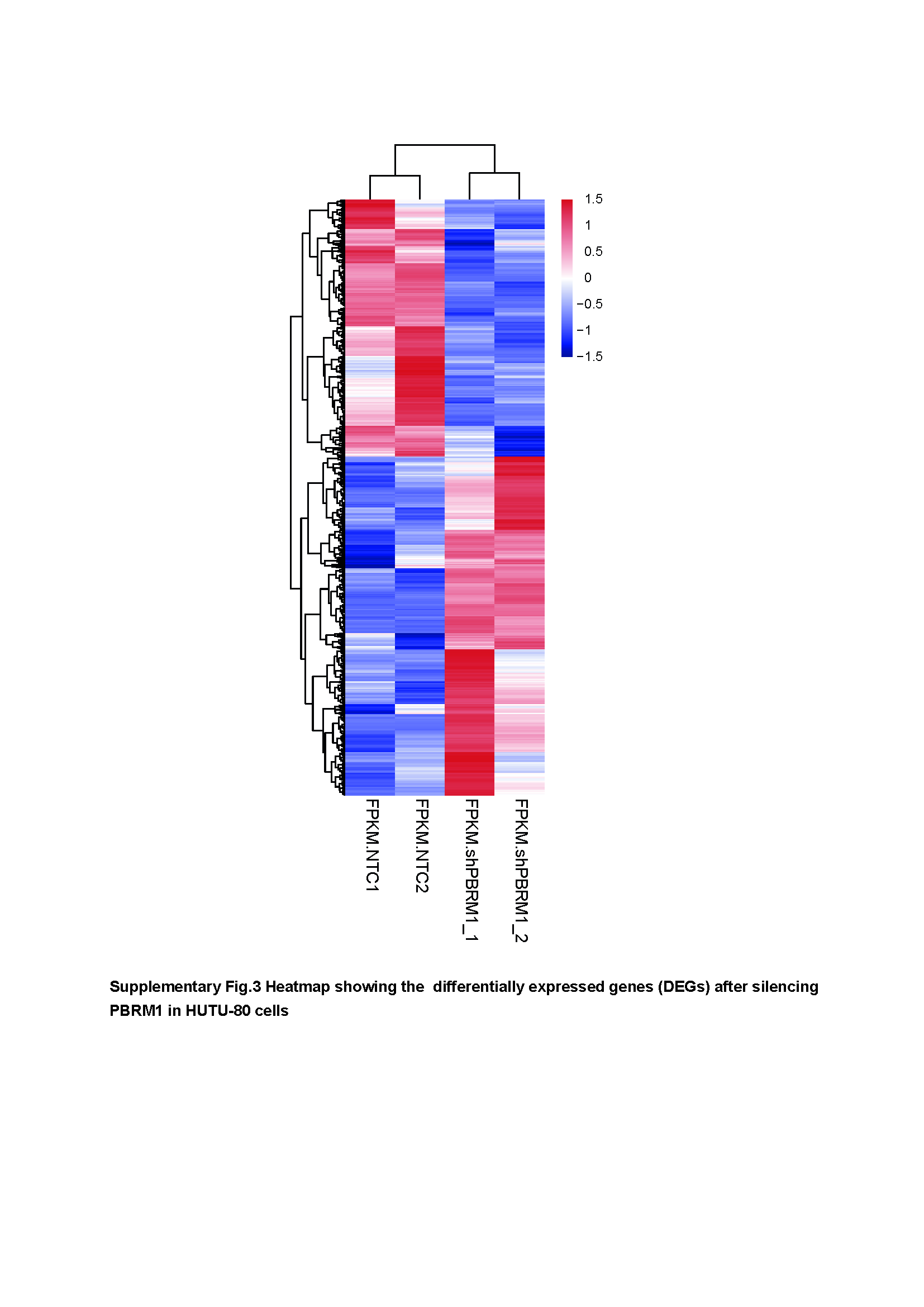

Supplement: Supplementary file 3 — Supporting Information [file CTM2-12-e1062-s004.tiff]

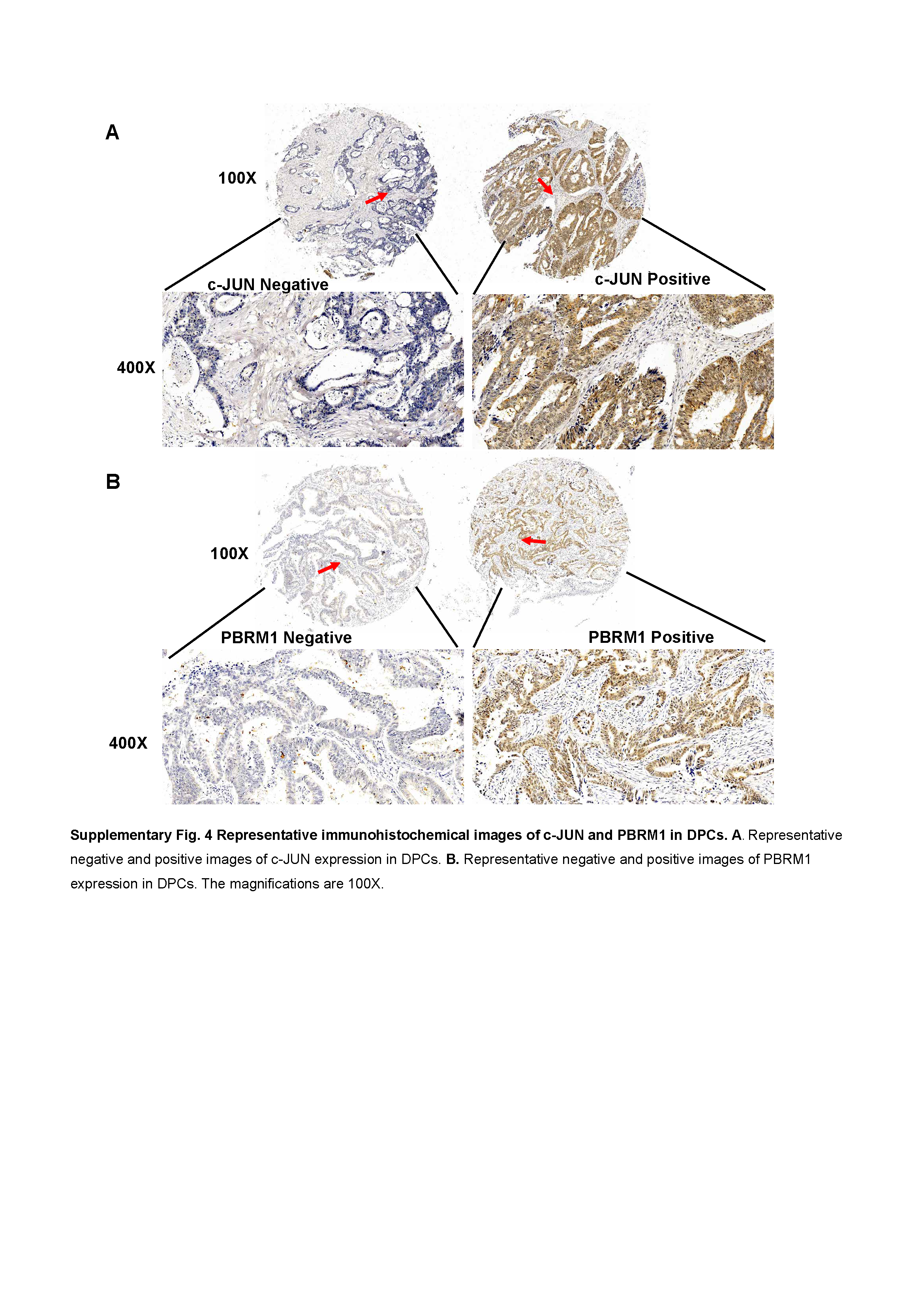

Supplement: Supplementary file 4 — Supporting Information [file CTM2-12-e1062-s002.tiff]
