# Supplementary material for: PBRM1 presents a potential prognostic marker and therapeutic target in duodenal papillary carcinoma
Source: Clin Transl Med. 2022 Sep 30;12(10):e1062. doi: 10.1002/ctm2.1062 (PMC9523678; doi:10.1002/ctm2.1062)
Supplement: Supplementary file 5 — Supporting Information [file CTM2-12-e1062-s003.docx]

**Method and Materials**

**Cell culture and treatments**

Human duodenal adenocarcinoma HUTU-80 cell and human breast cancer cell MDA-MB-231 were purchased from the Cell Resource Center of Chinese Academy of Medical Sciences. HUTU-80 cell were maintained in Minimum Essential Medium (MEM Eagles with Earle’s Balanced Salts, MEM-EBSS) with 10% fetal bovine serum (FBS) and 1% Nonessential amino acid (NEAA). MDA-MB-231 cells were cultured in 10% FBS DMEM medium. All mediums contain 1% antibiotics (100 units/ml penicillin and 100 μg/ml streptomycin) and all the cells were cultured in a humidified atmosphere in a 5% CO_2_ incubator at 37 °C.

**Generation of cell lines**

Knockdown was performed using shRNA-mediated knockdown with lentiviral construct pLKO.1 and packaged as Lentiviral by GUANZHOU RIBOBIO CO., LTD. The shRNA constructs contain the following mature antisense sequences: Human PBRM1 GTACCAAGATATTGACTCT. At the same time, the non-targeting sequence CAACAAGATGAAGAGCACCAA was selected for constructing a shNTC negative control. The full length of PBRM1 coding sequence was cloned into the pLVX-Puro vector for PBRM1 overexpression lentiviral construction. The empty pLVX-Puro lentiviral vector severed as corresponding negative control (NYC). The HUTU-80 cells were infected with concentrated virus at MOI=1 by using spin infection (1500 rpm in swing bucket centrifuge for 1 h) method. Fresh medium was added 24h after infection, and cells were allowed to selection for 2 weeks with 0.2 µg/ml of puromycin. The expression vector of pcDNA3.1-PBRM1, pcDNA3.1-c-Jun and empty controls were purchased from GUANZHOU RIBOBIO CO., LTD and stable transfected into HUTU-80 and MDA-MB-231 cells by using Lipofectamine 3000 and 600μg/ml of G418 selection. The efficiency of knockout and overexpression was confirmed by immunoblotting.

**Immunofluorescence (IF) staining**

Cells were washed twice with ice cold PBS then the cells were fixed using formalin for 20 minutes at room temperature. Next, cells were permeabilized with 0.5% Triton X-100 in PBS for 5 minutes at RT and washed 3 times with ice cold PBS. After the fixed cells were blocked with 10% goat serum for 1.5 hours, the cells were incubated overnight at 4°C with primary antibodies. The primary antibodies used were as follows: rabbit-PBRM1 (abcam; 1:200 in, 0.1% BSA, 0.01% Tween 20 in PBS) mouse anti-E cadherin (abcam; 1:500 in 0.1% BSA, 0.01% Tween 20 in PBS) and rabbit anti-VIM (abcam, 1:400 in 0.2% Triton X-100, 0.1% BSA, 0.05% Tween 20 in PBS). Cells were incubated with secondary antibody for 1 hour, followed by 3 washes at RT. Secondary antibodies were as follows: FITC goat anti-mouse and Cy3-conjugated anti-rabbit (Beyotime Biotechnology, China). Cell nuclei were counterstained with DAPI for 10 minutes and washed 3 times with PBS. Cells were incubated in PBS and imaged by Leica SP8 LIGHTNING confocal microscopy.

**5-Bromo-2-deoxyuridine (BrdU) staining assay**

For BrdU immunofluorescent staining, cells were grown on coverslips for 24 h, cells were incubated with 10 μg/ml BrdU (Sigma) for 30 min, then washed with phosphate-buffered saline (PBS) and fixed in 4% paraformaldehyde for 20 min. Subsequently, cells were blocked with 10% goat serum for 1 h, followed by a mouse primary monoclonal antibody against BrdU (1:1000, b8434, sigma, Germany) for 1 h and FITC goat anti-mouse IgG secondary antibody (Beyotime Biotechnology, China). DAPI solution (Beyotime Biotechnology, China) was used for nuclear staining and imaged by Leica SP8 LIGHTNING confocal microscopy.

**Trans-well and wound healing assay**

The migration and invasion assays were performed by a trans-well model as according to our previous studies^34, 35^. In brief, for migration assays, 5x10^4^ cells were resuspended in serum-free medium and seeded into the upper chamber. RPMI-1640 medium containing 20% FBS was added to the lower chamber as a chemoattractant. After 24 or 48 h, the cells were fixed and stained. Non‑invading cells in the upper chambers were removed with cotton swabs. The number of migrating or invading cells that had attached to the lower surface was then counted in 5 random fields under an Olympus IX71 microscope (x200 magnification, Olympus Corp.). Briefly, the migration assay was performed using Transwell plates containing membranes with 8 µm pores (3422; Corning Inc.). Cell invasion assays were performed using invasion chambers precoated with Matrigel (354480; BD Biosciences). The cells, which were knockdown or overexpression of PBRM1, or pretreated with 80 μM T5224, and the controls (2x10^5^ for invasion assays and 8x10^4^ cells for migration assays) were resuspended in serum-free medium and seeded into the upper chamber. RPMI-1640 medium containing 20% FBS was added to the lower chamber as a chemoattractant. After 24 or 48 h, the cells were fixed and stained. Non‑invading cells in the upper chambers were removed with cotton swabs. The number of migrating or invading cells that had attached to the lower surface was then counted in 5 random fields under an Olympus IX71 microscope (Olympus Corp. Japan).

For the scratch wound healing assay, 5 × 10^5^ cells/well were plated into a 6-well plate and incubated to reach confluence. The monolayer was scratched using a tip and washed with serum-free medium to remove detached cells. Then the cells were cultured in complete medium supplemented. The scratched wound was photographed under an Olympus IX71 microscope (Olympus Corp. Japan) at 0 h, 12 h and/or 24 h later.

**Western blot (WB)**

Briefly, the protein samples were subjected to SDS-PAGE and transferred to polyvinylidene difluoride (PVDF) membranes. Then, the membranes were blocked with 5% Bovine serum albumin (BSA) and incubrated with anti-PBRM1 (1:1000 dilution, abcam, USA), anti-E cadherin(1:2000 dilution, abcam, USA), anti-N cadherin(1:1000 dilution, abcam, USA), anti-cleaved PARP (1:1000 dilution, ET1608-10,Hangzhou Hua 'an Biotechnology Co. LTD), anti-PARP (1:1000 dilution, ET1608-56,Hangzhou Hua 'an Biotechnology Co. LTD), anti-cleaved caspase 3(1:1000 dilution, ET1602-47,Hangzhou Hua 'an Biotechnology Co. LTD), anti-caspase 3 (1:1000 dilution, ER30804,Hangzhou Hua 'an Biotechnology Co. LTD), anti-VIM(1:2000 dilution, abcam, USA), anti-p21(1:2000 dilution, abcam, USA), anti-p-c-Jun(s63) (1:500 dilution,sc-822, santcruz, USA), anti-γH2AX(1:500 dilution, sc-517348, santcruz, USA), anti-H2AX(1:500 dilution, sc-517336, santcruz, USA), anti-lamin B1(1:2000 dilution, ab16048, abcam, USA), anti-β-tubulin (1:5000 dilution, EM0103,Hangzhou Hua 'an Biotechnology Co. LTD) and anti-GAPDH (1:1000 dilution, ET1601-4,Hangzhou Hua 'an Biotechnology Co. LTD) antibodies. After incubation with the corresponding HRP-labeled secondary antibodies (1:10000, Hangzhou Hua 'an Biotechnology Co. LTD. China), the blots were developed with SuperSignal™ West Pico PLUS Chemiluminescent Substrate (34580, Thermo Scientific™) reagent and exposed in a Bio-Rad ChemiDoc™ MP System.

**Cell proliferation assay**

Cell proliferation was assessed using the Cell Proliferation CCK8 Assay kit (96992, Sigma-Aldrich, German) following the manufacturer's protocol. All cell lines were seeded into 96-well plates with 5x10^3^ cells in 200 µl culture medium per well. After attachment, 20 µl of CCK-8 reagents was added to each well every 24 h and then for an additional 4h incubation, the absorbance was measured at 450 nm using a Tecan Infinite 200 microplate reader (Tecan Group Ltd. Switzerland.)

**IHC Staining and immune signal evaluation**

Tissue sections were deparaffinized and then rehydrated with H2O. Antigen retrieval was performed by heating in a microwave oven at 95°C for 20 minutes in ethylenediaminetetraacetic acid buffer (pH 8.0). Endogenous peroxidase activity was blocked by incubating in 3% H2O2 for 5 minutes. Then the slides were incubated in primary antibodies as follows: overnight in a humidified chamber at 4°C for mouse monoclonal antibody c-Jun (sc-822, dilution 1:500, Santa Cruz, USA) and PBRM1(ab196022, dilution 1:800, Abcam, USA). Samples were then incubated with horseradish peroxidase–conjugated anti-rabbit/mouse secondary antibody of the DAKO REAL EnVision Detection System (K5007, DAKO, Denmark) for 30 minutes at room temperature. The immunoreaction was visualized by treatment with diaminobenzidine (DAB) chromogen for 5 minutes and then counterstaining with hematoxylin. Each slide was evaluated by two investigators who were blinded to any clinical information, and discrepancies were debated until a consensus was reached.

The immunoreactivity levels of PBRM1and c-Jun expression on the cell nucleus of each sample were estimated under a light microscope by the assessment of the average signal intensity (on a scale of 0–3). The proportion of cells that indicated positive staining (0, <5%; 1, 5–25%; 2, 26–50%; 3, 51–75%; 4, 76–100%) was independently estimated by two pathologists in the absence of clinical information, as described in our previous studies^34, 35^.The intensity and proportion scores were subsequently multiplied to obtain a composite score; the score of 0 to 3 was deﬁned as negative and a score of 4 to 12 as positive.

**X-ray irradiation**

A total of 5 × 10^6^ cells were plated into 10 cm cell culture dishes for attachment 24h, then were irradiated by 6-MV x-rays (600 MU/min, Trilogy System Linear Accelerator, Varian Medical Systems). The energy of the X-rays used to irradiate the cells was graded as 0, 2, 4 and 6 Gy. After irradiated, the cells were collected for performing cloning formation, IF or WB assays.

**Clonogenic assay**

Each group of cells were seeded into 6-well tissue culture plates and allowed to attach for 24h hours. Then change the fresh culture medium every 4 days for 3 weeks. In case of radiosensitivity measurements, cells were X-ray irradiated 24 h after plating. After two weeks of incubation, cultures were fixed and stained with crystal violet. Colonies formed with more than 50 cells were scored as survivors. Clonogenic fraction of irradiated cells was normalized to the plating efficiency of unirradiated controls.

**γH2AX foci assay**

Immunofluorescence staining of γH_2_AX were performed according the method described above. The following antibody was used at 1:500 anti-γH_2_AX (1:100 dilution, sc-517348, Santa Cruz, USA). Slides were then count staining with DAPI solution and images captured by using Leica SP8 LIGHTNING confocal microscopy.

For each treatment condition, foci were determined in at least 50 cells. The ImageJ (NIH) software was used to analyze the mean number of foci with combined area of γH2AX foci per nucleus.

**Proteome Profiler of Human Phospho-Kinase Array Kit**

Cells were collected and lysed in Cell Lysates buffer and pipetted up and down to resuspend and rock the lysates gently at 2-8 °C for 30 min, micro centrifuged at 14000g for 5 min, and the supernatant was transferred into a clean test tube. Sample protein concentrations were quantitated using a total protein assay of BCA method and then the extractions were aliquoted and stored at ≤-80℃. The Human Phospho-MAPK Array Kit ([ARY003B](https://www.rndsystems.com/cn/products/proteome-profiler-human-phospho-kinase-array-kit_ary003c), R&D Systems®, Inc. USA) was employed to detect the relative levels of phosphorylation of 43 human kinases. A total of 400µg of total protein of each sample were used in array membranes incubation and the array Detection steps were followed according to the manufacturer’s protocol ([ARY003B](https://www.rndsystems.com/cn/products/proteome-profiler-human-phospho-kinase-array-kit_ary003c), R&D Systems®, Inc. USA). The array membrane’s Chemiluminescence signal was detected using the Chemiluminescence Gel Imaging System (Bio-Rad, USA).

**Assay for transposase-accessible chromatin using sequencing（ATAC-seq）**

The ATAC-seq protocol was accorded and performed at the LC-BIO Technologies (Hangzhou) Co., LTD following the vendor’s recommended protocol^37^. Fifty thousands of HUTU-80-shNTC or HUTU-80-shPBRM1 cells were resuspended in cold Nuclei Lysis buffer (10Mm Tris-HCl, pH 7.4, 10mM NaCl, 3mM MgCl2 and 0.1% IGEPAL CA-630) for 5 minutes and centrifuged for 10 minutes at 500xg at 4 °C. Nuclei extraction was confirmed by microscopic inspection and the nuclei pellet was resuspended in the transposase reaction mix (25 μL 2x TD buffer, 2.5 μL Transposase (Illumina) and 22.5 μL of nuclease free water) for 30 minutes at 37 °C. After the transposition reaction, the sample was purified using a Qiagen Minelute kit and then amplified library fragments by using 1x NEBnext PCR master mix and 1.25 μM of custom Nextera PCR primers, using the following PCR conditions: 72°C for 5 minutes, 98°C for 30 seconds, followed by thermocycling at 98°C for 10 seconds, 63°C for 30 seconds and 72°C for 1 minute，Last extension at 72℃ for 5min.The libraries were purified using a Qiagen PCR cleanup kit yielding a final library. Then the high throughput sequencing was performed by HiSeq2500 using 50 bp paired end. After obtain the Raw reads, filtered to the joint , decontamination and mapping to the reference gene group, the High-quality mapped reads (MPAQ>=30) data was used for subsequent information analysis, the analysis process is as follows: 1) The Trimmomatic-0.35 Tool used for removing low-quality sequences. 2)The FastQC program used for Quality control analysis of the Clean data. 3）The Bowtie2 program used for aligning Clean data to the reference genome. 4）Calculate the genome coverage and correlation. 5）Peak-calling analysis on the genome using MACS2. 6）Peak screening with high confidence between samples using the IDR program. 7）Annotate Peaks and peak calling using the ChIPseeker package and macs2 callpeak Tools. 8）Motif analysis of combined peaks using the MEME program. 9）MAnorm program was used for each group difference Peaks analysis. 10) Peaks of differential accessibility were identified using the SICER-dfrb utility^38^ with a false discovery rate threshold of < 0.05 and a fold change threshold of > 1.5-fold difference in accessibility. 11) Scaled heat maps were generated for the peak regions using the computeMatrix and plotHeatmap utilities of deepTools.
