# Supplementary material for: PBRM1 presents a potential prognostic marker and therapeutic target in duodenal papillary carcinoma
Source: Clin Transl Med. 2022 Sep 30;12(10):e1062. doi: 10.1002/ctm2.1062 (PMC9523678; doi:10.1002/ctm2.1062)
Supplement: Supplementary file 7 — Supporting Information [file CTM2-12-e1062-s005.docx]

**Table 1 Relationship between** **clinicopathological factors, PBRM1 and c-Jun expression**

| **Clinical parameters** | **PBRM1** | | **t/χ2** | **P value** | **c-JUN** | | **t/χ2** | **P value** |
| --- | --- | --- | --- | --- | --- | --- | --- | --- |
|  | **Negative** | **Positive** |  |  | **Negative** | **Positive** |  |  |
| **Age (yrs)** | 62.63±11.32 | 63.24±11.36 | 0.284 | 0.777 | 62.87±11.96 | 62.93±10.64 | 0.029 | 0.977 |
| **Gender** |  |  |  |  |  |  | 0.902 | 0.342 |
| Male | 35(53.0%) | 26(52.0%) | 0.012 | 0.912 | 29(48.3%) | 32(57.1%) |  |  |
| Female | 31(47.0%) | 24(48.0%) |  |  | 31(51.7%) | 24(42.9%) |  |  |
| **Size** |  |  |  |  |  |  | 5.035 | 0.025 |
| ≥5cm | 34(51.5%) | 26(52.0%) | 0.003 | 0.959 | 25(41.7%) | 35(62.5%) |  |  |
| <5cm | 32(48.5%) | 24(48.0%) |  |  | 35(58.3%) | 21(37.5%) |  |  |
| **Differentiation** |  |  | 0.872 | 0.647 |  |  | 3.800 | 0.150 |
| Well | 6(9.1%) | 6(12.0%) |  |  | 8(13.3%) | 4(7.1%) |  |  |
| Moderately | 35(53.0%) | 29(58.0%) |  |  | 36(60.0%) | 28(50.0%) |  |  |
| Poorly | 25(37.9%) | 15(30.0%) |  |  | 16(26.7%) | 24(42.9%) |  |  |
| **Invasion Depth (T Grade)** |  |  | 6.952 | 0.073 |  |  | 26.231 | 0.000 |
| T1 | 10(15.2%) | 3(6.0%) |  |  | 9(15.0%) | 4(7.1%) |  |  |
| T2 | 15(22.7%) | 13(26.0%) |  |  | 23(38.3%) | 5(8.9%) |  |  |
| T3 | 5(7.6%) | 11(22.0%) |  |  | 11(18.3%) | 5(8.9%) |  |  |
| T4 | 36(54.5%) | 23(46.0%) |  |  | 17(28.3%) | 42(75.0%) |  |  |
| **Lymphatic Metastasis**  **(N Grade)** |  |  | 0.033 | 0.857 |  |  | 0.228 | 0.633 |
| No | 42(63.6%) | 31(62.0%) |  |  | 39(65.0%) | 34(60.7%) |  |  |
| Yes | 24(36.4%) | 19(38.0%) |  |  | 21(35.0%) | 22(39.3%) |  |  |
| **Distant metastasis (M Grade)** |  |  | 0.120 | 0.729 |  |  | 0.417 | 0.518 |
| M0 | 64(97.0%) | 49(98.0%) |  |  | 59(98.3%) | 54(96.4%) |  |  |
| M1 | 2(3.0%) | 1(2.0%) |  |  | 1(1.7%) | 2(3.6%) |  |  |
| **TNM Stages** |  |  | 0.558 | 0.906 |  |  | 13.779 | 0.003 |
| Ⅰ | 22(33.3%) | 14(28.0%) |  |  | 27(45.0%) | 9(16.1%) |  |  |
| Ⅱ | 20(30.3%) | 17(34.0%) |  |  | 12(20.0%) | 25(44.6%) |  |  |
| Ⅲ | 22(33.3%) | 18(36.0%) |  |  | 20(33.3%) | 20(35.7%) |  |  |
| Ⅳ | 2(3.0%) | 1(2.0%) |  |  | 1(1.7%) | 2(3.6%) |  |  |
| **Lymphatic invasion** |  |  | 0.011 | 0.915 |  |  | 3.828 | 0.050 |
| Yes | 7(10.6%) | 5(10.0%) |  |  | 3(5.0%) | 9(16.1%) |  |  |
| No | 59(89.4%) | 45(90.0%) |  |  | 57(95.0%) | 47(83.9%) |  |  |
| **Vascular invasion** |  |  | 0.465 | 0.495 |  |  | 5.200 | 0.023 |
| Yes | 21(31.8%) | 13(26.0%) |  |  | 12(20.0%) | 22(39.3%) |  |  |
| No | 45(68.2%) | 37(74.0%) |  |  | 48(80.0%) | 34(60.7%) |  |  |
| **Nerve invasion** |  |  | 4.458 | 0.035 |  |  | 10.175 | 0.001 |
| Yes | 22(33.3%) | 8(16.0%) |  |  | 8(13.3%) | 22(39.3%) |  |  |
| No | 44(66.7%) | 42(84.0%) |  |  | 52(86.7%) | 34(60.7%) |  |  |
| **PBRM1 expression** |  |  |  |  |  |  | 32.259 | 0.000 |
| Negative |  |  |  |  | 19(31.7%) | 47(83.9%) |  |  |
| Positive |  |  |  |  | 41(68.3%) | 9(16.1%) |  |  |
