# Supplementary material for: PBRM1 presents a potential prognostic marker and therapeutic target in duodenal papillary carcinoma
Source: Clin Transl Med. 2022 Sep 30;12(10):e1062. doi: 10.1002/ctm2.1062 (PMC9523678; doi:10.1002/ctm2.1062)
Supplement: Supplementary file 8 — Supporting Information [file CTM2-12-e1062-s001.docx]

Original research

**PBRM1 presents a potential prognostic marker and therapeutic target in** **duodenal papillary carcinoma**

Xujun He^1^^,2,3#^, Ji Xu^1,3#^, Nan Niu^4#^, Guoxi Xu^5#^, Honglin Zhu^6#^, Zhengchuang Liu^1^, Yiping Mou^1,3^, Zhengyuan Qian^1,3^, Huiju Wang^1,3^, Junfeng Hu^1,3^, Tonghui Ma^6^, Jie Ma^7^, Houquan Tao^1,3*^

**Supplementary Materials:**

Supplementary Methods: 1

Supplementary Table: 1

Supplementary Sheet: 1

Supplementary Figure: 4

**Note:** the supplementary sheets and figures are uploaded as separate files.

**Supplementary Methods 1. The detailed Materials and Methods**

**Supplementary Table 1. Relationship between clinicopathological factors, PBRM1 and c-JUN expression**

**Supplementary Sheet 1. The clinicopathological features of 15 DPCs**

**Supplementary Fig. 1 PBRM1 expression inhibited the proliferation, colony formation, migration, and invasion abilities of HUTU-80 cells and MDA-MB-231 cells.** A, PBRM1 mutation lollipops in the MDA-MB231 cell line. B-E, PBRM1 expression inhibited the proliferation, colony formation, migration, invasion and wound-healing abilities of HUTU-80 cells and MDA-MB-231 cells. (*P < 0.05).

**Supplementary Fig. 2 Knockdown of PBRM1 by shRNA #2 promote HUTU-80 cell progress and epithelialmesenchymal transformation. A,** IF result of PBRM1 expression in HUTU-80-shNTC and HUTU-80-shPBRM1#2 cells. B. Downregulation of PBRM1 expression resulted in an increase in colony formation in HUTU-80-shPBRM1#2 cells. C. Downregulation of PBRM1 expression resulted in an increase in proliferation in HUTU-80 cell (MTs method). D. Downregulation of PBRM1 expression resulted in an increase migration and invasion ability in HUTU-80-shPBRM1#2 cells. E. IF result of VIM, PBRM1, N-cadherin ,E-cadherin and p-c-jun expression in HUTU-80-shNTC and HUTU-80-shPBRM1#2 cells. F. WB analysis of PBRM1, VIM,N-cadherin , c-JUN, p-c-jun and E-cadherin expression in HUTU-80-shNTC and HUTU-80-shPBRM1 #2 cells.

**Supplementary Fig. 3 Heatmap showing the differentially expressed genes (DEGs) after silencing PBRM1 in HUTU-80 cells.**

**Supplementary Fig. 4 Representative immunohistochemical images of c-JUN and PBRM1 in DPCs. A.** Representative negative and positive images of c-JUN expression in DPCs. **B.** Representative negative and positive images of PBRM1 expression in DPCs. The magnifications are 100X.
